# Spatial single-cell profiling identifies protein kinase Cδ-expressing microglia with anti-tumor function in glioblastoma

**DOI:** 10.1016/j.isci.2025.114281

**Published:** 2025-11-29

**Authors:** Reza Mirzaei, Reid McNeil, Charlotte D'Mello, Britney Wong, Susobhan Sarkar, Frank Visser, Candice Poon, Pinaki Bose, V. Wee Yong

**Affiliations:** 1 Department of Oncology, Cross Cancer Institute, University of Alberta, Edmonton, AB, Canada; 2 Arnie Charbonneau Cancer Institute, University of Calgary, Calgary, AB, Canada; 3 Department of Biochemistry and Molecular Biology, University of Calgary, Calgary, AB, Canada; 4 Department of Clinical Neurosciences, University of Calgary, Calgary, AB, Canada; 5 Hotchkiss Brain Institute, University of Calgary, Calgary, AB, Canada; 6 Department of Oncology, University of Calgary, Calgary, AB, Canada

**Keywords:** Components of the immune system, Cancer, Transcriptomics, Model organism

## Abstract

Glioblastoma (GBM) contains diverse immune and tumor cell populations whose spatial organization influences disease progression. To better understand how immune cells interact with brain tumor-initiating cells (BTICs), we applied integrated single-cell and spatial transcriptomic approaches to map the immune landscape in a GBM mouse model. This analysis revealed a distinct subset of microglia expressing protein kinase Cδ (PKCδ) that localizes near BTIC-rich regions and displays features associated with anti-tumor activity. We validated the presence of PKCδ^+^ microglia in human GBM tissues and found that PKCδ enhances inducible nitric oxide synthase (iNOS) expression, supporting microglial cytotoxic and phagocytic functions. Increasing PKCδ levels in microglia, either through adeno-associated viral delivery or niacin treatment, strengthened their ability to engulf and kill BTICs. Analysis of patient datasets further showed that higher PKCδ expression associates with immune activation and cell death pathways. These findings identify PKCδ^+^ microglia as a therapeutically relevant component of the GBM microenvironment.

## Highlights

• Spatial heterogeneity shapes diverse cell interactions in the GBM microenvironment
• Single-cell spatial profiling identifies anti-tumor PKCδ^+^ microglia in GBM
• PKCδ activation boosts microglial phagocytosis and killing of BTICs
• Increasing PKCδ reprograms microglia toward an anti-tumor phenotype

## Graphical abstract

**Figure:**
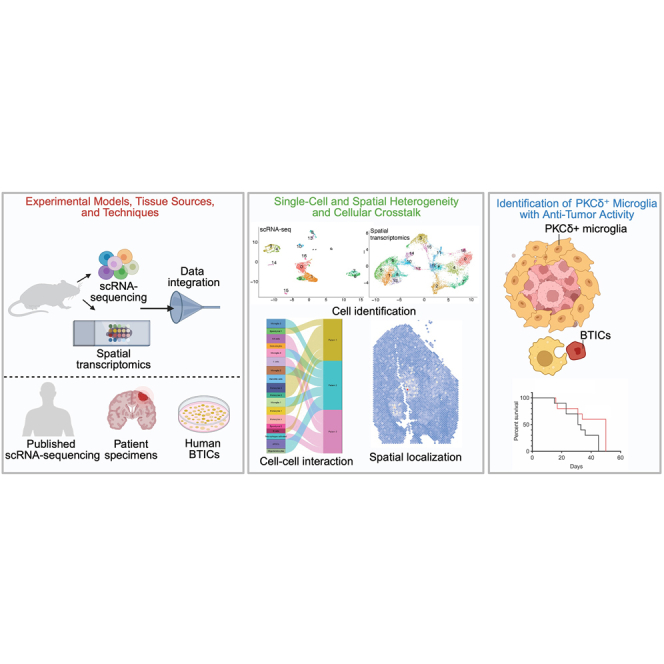

## Introduction

Glioblastoma (GBM) is the most common primary malignant brain tumor in adults.^1^ The standard treatment consists of maximal surgical resection, followed by radiotherapy and chemotherapy with the oral alkylating agent temozolomide.^2^ Despite these aggressive treatments—along with adjunct therapies such as tumor-treating fields (TTFields)—the median overall survival remains under 21 months.^3^ A major challenge in improving GBM outcomes lies in the complex tumor microenvironment (TME) of the brain. The brain TME is characterized by two key features: its highly immunosuppressive environment and profound intratumoral heterogeneity, both of which play a significant role in the poor outcomes associated with GBM and in introducing new effective treatment.^4^^,^^5^^,^^6^^,^^7^

The brain TME has a unique cellular composition, featuring a diverse population of specialized resident cells.^8^ Resident microglia are one of the first immune cells to encounter tumor cells,^9^ but they have been understudied, often being mixed with monocyte-derived macrophages (MDMs) in many studies. Microglia possess intrinsic anti-tumor functions, such as phagocytosis of tumor cells.^10^^,^^11^^,^^12^ However, various studies, including our own,^13^^,^^14^^,^^15^ have demonstrated that these anti-tumor functions are suppressed within the brain TME. Notably, microglia interact with brain tumor-initiating cells (BTICs) - a subpopulation that drives tumorigenesis and exhibits higher therapy resistance than differentiated GBM cells.^16^^,^^17^^,^^18^ Therefore, studying the interactions between microglia and BTICs is essential for understanding tumor progression and anti-tumor immunity.

Single-cell RNA-sequencing (scRNA-seq) reveals that both microglia and BTICs exhibit intratumoral heterogeneity.^5^^,^^19^^,^^20^ Additionally, emerging technologies such as spatial transcriptomics demonstrate that various types of microglia and BTICs are distributed across distinct regions of the brain TME.^20^^,^^21^^,^^22^ However, a key yet understudied question is how these diverse populations communicate across spatial domains. Understanding this communication, while accounting for both cellular and spatial heterogeneity, is crucial as it could reveal new strategies to reactivate the anti-tumor functions of microglia and inhibit the growth of tumor cells such as BTICs.

To address this knowledge gap, we applied integrative single-cell and spatial transcriptomics in a syngeneic mouse model of GBM, enabling the comprehensive characterization of tumor and immune cell heterogeneity with a focus on microglia-BTIC interactions. To ensure translational relevance, we validated our findings using publicly available human GBM scRNA-seq datasets, patient-derived BTICs, and clinical specimens. Our study uncovers a spatially restricted subpopulation of microglia expressing protein kinase Cδ (PKCδ) with anti-tumor activity. These PKCδ^+^ microglia exhibit enhanced phagocytosis of BTICs and can be expanded by the repurposed drug niacin, resulting in suppressed GBM growth.

## Results

### Single-cell and spatially resolved the transcriptomic profiling of the glioblastoma microenvironment

**Figure 1:**
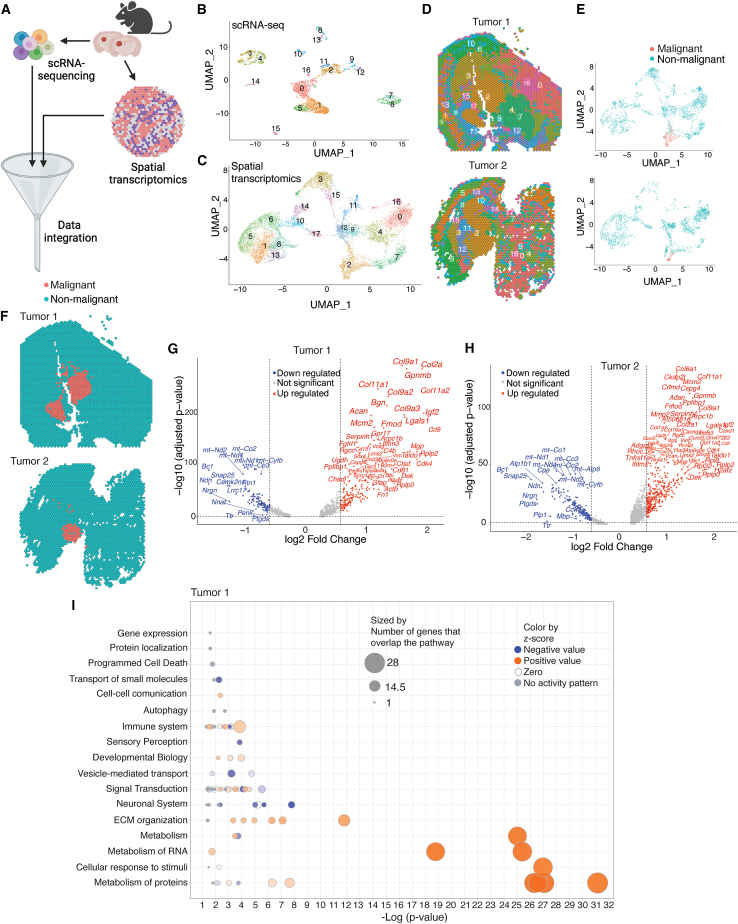
Single-cell and spatially resolved the transcriptomic profiling of the GBM TME (A) Schematic representation of the integrated scRNA-seq and spatial transcriptomics analysis conducted in the GBM mouse model. (B and C) UMAP plots displays Seurat clusters identified through scRNA-seq of mouse GBM (B) and spatial transcriptomics analysis (C). (D) Spatial Dimplot of Seurat clusters overlaid on tissue images. (E) UMAP visualization of spatial clusters annotated as malignant or non-malignant based on copy number variation (CNV) analysis. (F) Spatial distribution of malignant and non-malignant regions in two mouse tumors (Tumor 1 and Tumor 2). (G and H) Volcano plots illustrates the differential expression of genes between malignant and non-malignant areas in two mouse tumors. (I) Canonical pathways enriched in DEGs between malignant and non-malignant regions in mouse tumor 1.

To map the transcriptomic landscape of the brain TME, we paired scRNA-seq with spatial transcriptomics in a syngeneic mouse model of GBM (Figure 1A). The model was generated by intracranially implanting BTICs from NPcis mice—bearing Nf1 and Trp53 mutations^23^—into C57BL/6 recipients, a model previously shown to mirror key features of human GBM.^24^ After confirming tumor growth by bioluminescence imaging four weeks after implantation (Figure S1A), brains from five mice were harvested and pooled (Figure S1B). Single-cell suspensions were prepared, and leukocytes were enriched using a Percoll gradient. Live cells were then purified by flow cytometry with a live/dead discrimination dye and subjected to scRNA-seq (10× Genomics).

Initial quality control yielded 5,440 high-quality single cells. Unsupervised clustering based on gene expression signatures identified 17 clusters (Figure 1B). Deconvolution assigned these clusters to 11 canonical cell populations: acute neural stem cells (aNSCs), B cells, dendritic cells (DCs), erythrocytes, granulocytes, monocyte-derived macrophages (MDMs), microglia (MG), natural killer cells, neurons, oligodendrocytes, and T cells (Figure S1C). Inferred copy number variation (inferCNV) analysis did not reveal CNVs at cancer-associated loci—such as amplifications on chromosomes 6, 7, or 11, or deletions on chromosomes 9, 12, or 19 (Figure S1D)—confirming the absence of tumor cells in the scRNA-seq dataset. To further resolve macrophage heterogeneity, we performed a higher-resolution deconvolution. Trajectory analysis subdivided monocytes and microglia into distinct cellular states (Figures S1E–S1H), resulting in five monocyte subsets and four microglia subsets. This refinement expanded the dataset to 21 distinct cell-type clusters, which were used for downstream analyses (Figure S1I).

We analyzed brain tissue from two additional tumor-bearing mice and a tumor-free control using Visium spatial transcriptomics, following our published workflow.^20^ After initial quality control—removing genes with average expression below 0.1—we further assessed spot quality using three metrics: number of counts, number of features, and percentage of mitochondrial reads. More than 80% of spots passed quality control (Figures S2A–S2D). Following normalization and batch correction, Seurat partitioned the 12,885 captured spots into 18 distinct clusters (Figures 1C, 1D, S3A, and S3B). To identify tumor regions, we used InferCNV to detect CNVs. Cluster 2 showed a strong enrichment for CNVs—most prominently the chromosome-7 gains typical of GBM^21^—relative to all other clusters (Figures 1E, S3C, and S3D). Spatial mapping placed Cluster 2 within the tumor core (Figure 1F), coinciding with the highly proliferative areas seen on H&E sections in our earlier study.^20^

Differential expression analysis (DEA) comparing InferCNV-defined tumoral spots with non-tumoral regions revealed distinct transcriptional profiles. Volcano plots showed significant up-regulation of cancer-associated genes, including extracellular matrix (ECM) components (*Col6a1*, *Col2a1*, *Bgn*, *Acan*, *Cspg4*, *Vcan*, *Mgp*, *Mmp2*), cell-cycle regulators (*Ccnd3*, *Cdk4*, *Ccnd1*), immune-related genes (*Ifitm2*, *Ifitm3*, *B2m*, *Lgals1*, *H2-D1*, *Tnfrsf1a*, *Cd9*, *Gpnmb*), and the complement component *C4b* in tumoral spots (Figures 1G, 1H, and S3E–S3H). Ingenuity pathway analysis (IPA) of cluster 2 differentially expressed genes (DEGs) from each mouse revealed significant enrichment in pathways related to metabolism, ECM organization, the neuronal system, immune responses, and cell-cell communication (Figures 1I and S4A).

To evaluate the spatial relevance of cluster 2 DEGs in human GBM, we analyzed 44,712 cells from a published scRNA-seq dataset comprising seven patients with GBM.^5^ Using our previously established deconvolution approach^20^ (Figure S4B), we mapped the expression profiles of mouse tumoral spot genes (cluster 2) to human GBM cells at single-cell resolution. ECM genes—*COL6A1*, *BGN*, *CSPG4*, *VCAN,* and *MGP*—were expressed primarily by malignant cells (Figure S4C). In contrast, immune-related genes, including *IFITM2*, *IFITM3*, *LGALS1*, *TNFRSF1A*, *CD9,* and *GPNMB,* were enriched in microglia and MDMs. These expression patterns highlight the distinct transcriptomic landscapes of macrophages versus tumor cells and suggest their divergent contributions to GBM progression.

### Integrative single-cell spatial transcriptomics uncovers cellular localization in the glioblastoma microenvironment

**Figure 2:**
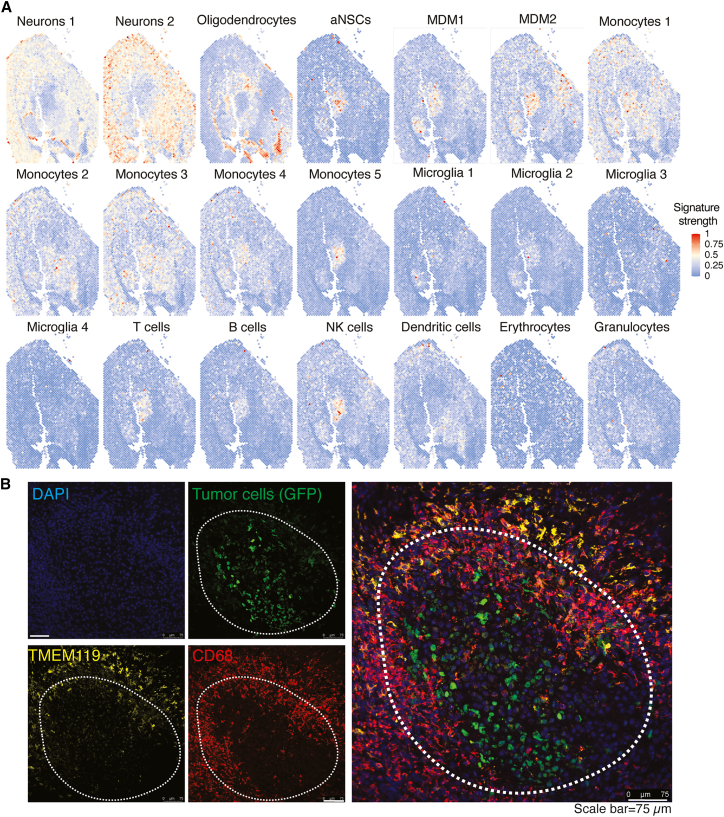
Single-cell spatial heterogeneity in the GBM microenvironment (A) Deconvoluted cell type composition in spatial transcriptomics in mouse Tumor 1. (B) Representative immunofluorescence (IF) image of a mouse brain implanted with syngeneic tumor cells. Scale bars: 75 μm.

To map the spatial distribution of cell types in the GBM microenvironment, we integrated our single-cell and spatial transcriptomic datasets. Using the mouse single-cell data as a reference, we performed spatially resolved cell-type deconvolution, which uncovered distinct localization patterns for stromal and immune populations. Neuronal gene expression signatures were distributed throughout the tissue but notably absent from defined tumor regions (Figures 2A and S5). Oligodendrocyte signatures were enriched in non-tumoral areas, whereas aNSC-associated signatures were confined to the tumor core, suggesting a potential role for aNSCs in tumor progression.

To refine the spatial findings, we performed gene set enrichment analysis (GSEA) on macrophage subsets using MSigDB pathways in conjunction with spatial localization data. Monocyte subsets 1–5 were detected in both tumor and non-tumor regions but primarily clustered at the tumor margins (Figures 2A and S5). Notably, subset 5 preferentially infiltrated the tumor core and was negatively enriched for numerous immune pathways critical to anti-tumor defense—including adaptive and innate immune responses, IFN-γ/IFN-α signaling, MHC class II antigen presentation, TNF signaling, and other cytokine pathways (Figures S6 and S7)—suggesting a tumor-supportive phenotype. In contrast, this subset was positively enriched for cell cycle-related genes, indicating active proliferation.

Within the MDM compartment, subtypes MDM1 and MDM2 were consistently enriched in tumor regions relative to non-malignant tissue (Figures 2A and S5). GSEA revealed negative enrichment of IFN and broader cytokine-signaling pathways in both subtypes (Figure S8), indicating an immunosuppressive profile. Among the four microglia clusters, MG1 and MG2 preferentially accumulated in tumor areas, whereas MG3 and MG4 were spread across malignant and non-malignant zones (Figures 2A and S5). Notably, MG2 showed positive enrichment for immune-activation pathways—including MHC-II antigen presentation, IFN-α/γ signaling, and broader cytokine signaling—suggesting an anti-tumor phenotype (Figures S9 and S10).

Lymphocytes (T, B, and NK cells) likewise concentrated in the tumor core, whereas DCs and granulocytes displayed a more uniform distribution; the tumor core was largely devoid of DCs (Figures 2A and S5). Given the pivotal roles of MDMs and microglia in tumor progression and immunity, we validated their spatial distribution at the protein level. Immunofluorescence (IF) staining for CD68 and TMEM119 on brain sections from an independent cohort confirmed that CD68^+^TMEM119^-^ MDMs densely populated the tumor core, while CD68^+^TMEM119^+^ microglia were enriched at the tumor periphery (Figure 2B).

### Heterogeneous cellular interactions in the glioblastoma microenvironment

**Figure 3:**
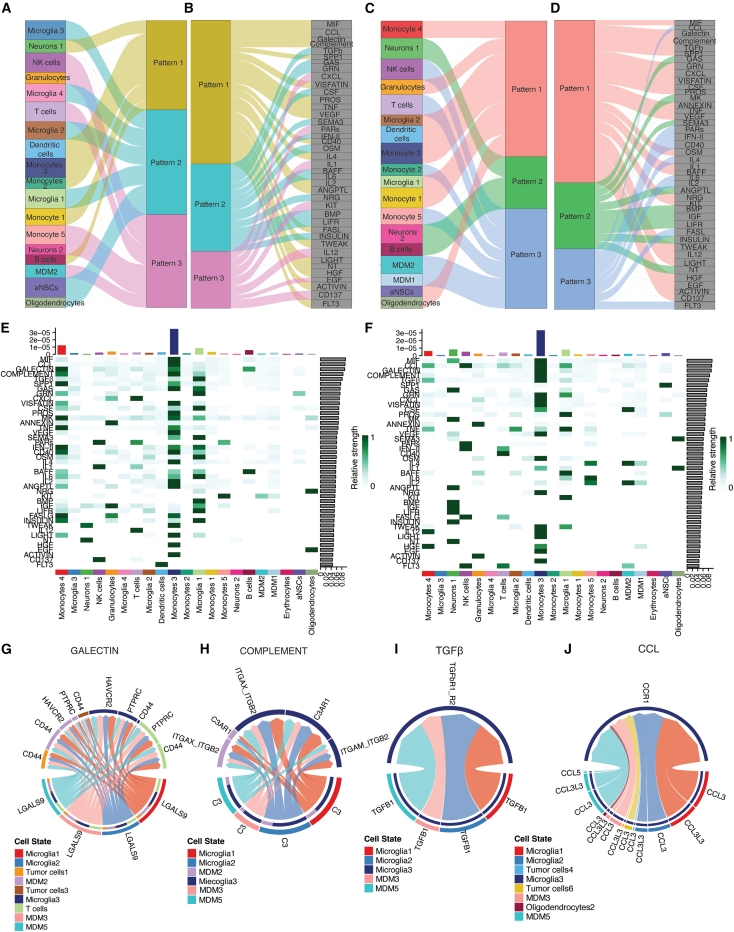
Heterogeneous cellular interactions in the brain TME (A) River plot illustrates patterns of incoming communication among target cell populations based on the scRNA-seq data from the mouse GBM model. (B) Signaling networks enriched within three distinct incoming communication patterns identified in the mouse GBM microenvironment. (C) River plot displays patterns of outgoing communication from secreting cell populations in mouse GBM. (D) Signaling networks enriched within three distinct outgoing communication patterns. (E and F) Heatmaps show signaling pathways that contribute significantly to incoming (E) or outgoing (F) communication across specific cell groups in mouse GBM. (G–J) Chord diagrams represent significant ligand-receptor interactions between source and target cell populations in human GBMs.

We extended our analysis to map cell-cell communication and clustered the incoming signaling inputs to scRNA-seq-defined populations into three distinct patterns (Figure 3A). Pattern 1—shared by neuron 1, DCs, monocyte subsets 1–3, and B cells—was enriched for macrophage migration inhibitory factor (MIF), chemokine ligand (CCL), galectin, complement, secreted phosphoprotein 1 (SPP1), protein S (PROS), visfatin, TNF, vascular endothelial growth factor (VEGF), CD40, IL-4, IL-2, and FAS-ligand networks. Pattern 2, which characterized microglia clusters MG1–MG4, aNSCs, and oligodendrocytes, featured signals from TGF-β, growth-arrest-specific protein (GAS), progranulin (GRN), colony-stimulating factors (CSFs), IFN-II, oncostatin M (OSM), B-cell-activating factor (BAFF), IL-6, leukemia inhibitory factor receptor (LIFR), and insulin pathways. Pattern 3, defining NK cells, granulocytes, T cells, monocyte subset 5, neuron 2, and MDM2, was dominated by CXCL chemokines, protease-activated receptors (PARs), IL-1, KIT, IL-12, epidermal growth factor (EGF), and CD137 networks (Figure 3B).

Outgoing communication is also segregated into three distinct patterns (Figure 3C). Pattern 1—shared by monocyte 4, granulocytes, MG2, DCs, monocyte 3, MG1, monocyte 1, aNSCs, and oligodendrocytes—exported a broad repertoire of signals, including MIF, galectins, complement, TGF-β, SPP1, GRN, CXCL chemokines, visfatin, CSFs, annexins, and TNF (Figure 3D). Pattern 2, used by neurons 1 and 2 and B cells, relied on GAS, midkine, ANGPTL, IGF, LIFR, and insulin pathways. Pattern 3, characteristic of NK cells, T cells, monocytes 2 and 5, MDM1 and 2 was dominated by CCL chemokines, PARs, type-II IFN, CD40, IL-4, IL-6, IL-2, FAS-ligand, and FLT3 signaling. Together, these distinct export programs underscore the diverse strategies by which each cell type transmits information within the GBM microenvironment.

Monocyte subsets 3 and 4 and MG1—the immune populations most enriched within tumor regions—received the strongest incoming signals via tumor-promoting networks such as galectin, complement, TGF-β, SPP1, TNF, and IL-4 (Figure 3E). These same infiltrating cells also exported the highest levels of CCL, GRN, CXCL, TNF, IL-4, IL-6, IL-2, and IL-12 signals (Figure 3F). In contrast, MG3, MG4, and monocyte subsets 1 and 2—which were less concentrated within tumor cores—exhibited significantly weaker engagement with pro-tumor or immunosuppressive pathways, including MIF, TNF, IL-4, IL-6, OSM, galectin, and TGF-β. Collectively, these findings reveal that spatially distinct immune subsets in GBM display divergent interaction profiles, with MIF, galectin, complement, TGF-β, and CCL signaling emerging as dominant mediators of cell-cell communication within the GBM microenvironment (Figure S11).

To translate our findings to human GBM, we analyzed a published human scRNA-seq dataset,^5^ and focused on ligand-receptor interactions within the five signaling networks highlighted in the mouse study—MIF, galectin, complement, TGF-β, and CCL. All networks except MIF were enriched in the human TME (Figures 3G–3J). In the galectin axis, *LGALS9* was highly expressed by MG1, MG2, MDM3, and MDM5, enabling crosstalk with T cells, MG3, MDM2, and tumor-cell clusters 1 and 3 through the receptors *CD44*, *PTPRC*, and *HAVCR2* (Figure 3G). Within the complement pathway, MG1, MG2, MDM3, and MDM5 secreted *C3*, which signaled to MG3 and MDM2 via *ITGAM*–*ITGB2*, *ITGAX*–*ITGB2*, and *C3AR1* (Figure 3H). TGF-β signaling connected MG1, MG2, MDM3, and MDM5 to MG3 through the *TGFBR1*/*2* receptor pair (Figure 3I). Finally, in the CCL network, *CCL3* and *CCL3L3* produced by MG1, MG2, tumor-cell clusters 4 and 6, oligodendrocytes, and MDM5 engaged *CCR1* on MG3, while *CCL5*—expressed exclusively by MDM5—also targeted MG3 (Figure 3J).

### Identification of protein kinase Cδ-positive microglia in the glioblastoma microenvironment

**Figure 4:**
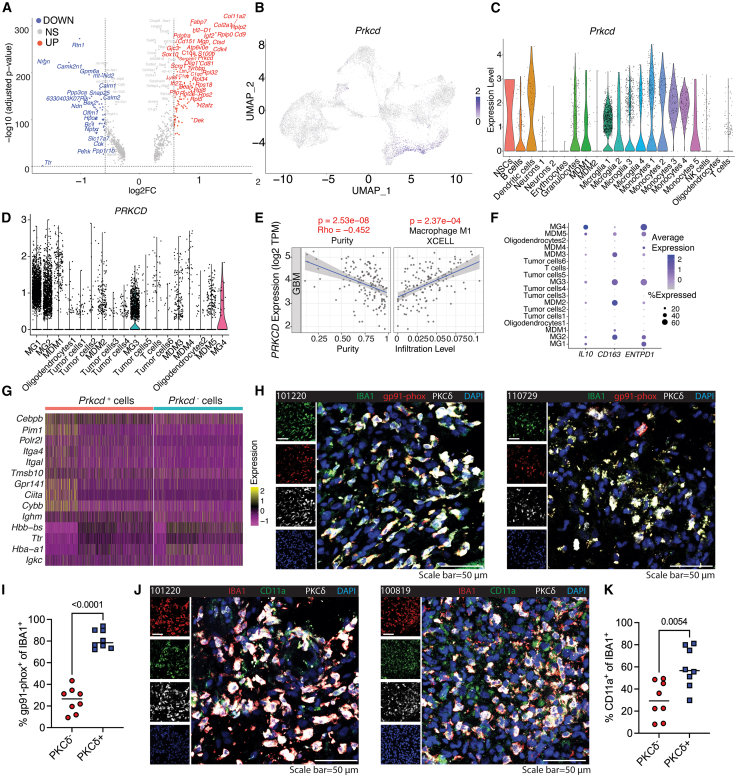
Identification of PKCδ-positive microglia in the GBM microenvironment (A) Volcano plot displays differentially expressed genes (DEGs) between cluster 2 and all other clusters in mouse spatial transcriptomics. (B) UMAP plot shows *Prkcd* expression across spatial transcriptomics data in the mouse GBM model. (C and D) Violin plots show *Prkcd* expression in mouse scRNA-seq clusters (C) and *PRKCD* expression in human GBM scRNA-seq clusters (D). (E) Spearman’s correlation analysis of *PRKCD* expression with tumor purity and macrophage phenotype using the Tumor Immune Estimation Resource (TIMER2.0). (F) Dot plot illustrates the expression of M2-like macrophage markers in human GBM scRNA-seq data. (G–I) (G) Heatmap of DEGs between *Prkcd*^+^ and *Prkcd*^−^ macrophage populations (monocytes, MDMs, and microglia) in mouse GBM scRNA-seq data. (H, I) Representative immunofluorescence (IF) images (H) and quantification (I) of IBA1^+^, PKCδ^+^, and gp91-phox (*CYBB*)^+^ cells in human GBM specimens from eight patients. (J and K) Representative IF images (J) and quantification (K) of IBA1^+^, PKCδ^+^, and CD11a (*ITGAL*)^+^ cells in human GBM specimens from eight patients. Data in panels I and K are presented as mean ± SEM and were analyzed using a two-tailed unpaired *t* test. Scale bars on IF images: 50 μm.

To better characterize immune modulators enriched in tumor regions, we analyzed the spatial transcriptomic data and identified several genes associated with immune regulation—including *H2-D1*, *C1qa*, *C1qc*, *Ly86*, *Ifi27*, and *Prkcd* (encoding PKCδ)—that were significantly upregulated in tumor-associated spots (Figure 4A). Because the role of PKCδ in the TME is still poorly understood, we centered our subsequent analyses on PKCδ. Its expression was elevated in both cluster 2 (tumor core) and cluster 7 (tumor-adjacent margin) (Figures 4B and S3H), areas where infiltrating immune cells can influence tumor control. In mouse scRNA-seq data, *Prkcd* was highest in microglia, monocytes, and MDMs, with peak levels in MG4, MG3, MG2, and monocyte 1 (Figure 4C). Spatial maps confirmed that these *Prkcd*-expressing populations infiltrated both tumor and peritumoral tissue, with pronounced enrichment at the tumor border (Figure 1D). A parallel analysis of human GBM scRNA-seq datasets revealed higher *PRKCD* expression in microglia than in other clusters, following an MG1 > MG2 > MG3 expression pattern (Figure 4D).

Analysis of The Cancer Genome Atlas (TCGA) RNA-seq data with TIMER2.0^25^^,^^26^ showed that *PRKCD* expression was inversely correlated with tumor purity and positively associated with M1-like, anti-tumor macrophages in GBM (Figure 4E). In human GBM single-cell data, the MG1 cluster displayed a less immunosuppressive (M2-like) profile than other clusters, evidenced by lower *IL10*, *CD163*, and *ENTPD1* expression (Figure 4F). Ligand-receptor analysis likewise revealed that MG1 generated and received fewer immunosuppressive signals—including *IL10*, *IL16*, *IL6*, *GPNMB*, and *MIF*—than other immune populations (Figure S12A).

To refine the profile of PKCδ-expressing macrophages, we compared *Prkcd*^+^ and *Prkcd*^−^ microglia, monocytes, and MDMs in the mouse scRNA-seq dataset. *Cybb* emerged as one of the most up-regulated genes in the *Prkcd*^+^ subsets (Figure 4G). A human GBM scRNA-seq cohort showed *CYBB* expression peaked in MG1 and declined through MG2 and MG3 (Figure S12B), paralleling the pattern of *PRKCD* expression (Figure 4D). *CYBB* encodes gp91-phox, the catalytic subunit of phagocyte NADPH oxidase that generates superoxide and other reactive oxygen species essential for antimicrobial activity.^27^ We validated these transcriptomic findings by IF staining for PKCδ, gp91-phox, and the macrophage marker IBA1 on surgical GBM specimens (*n* = 8), observing significantly higher gp91-phox levels in PKCδ^+^ macrophages than in PKCδ^−^ counterparts (Figures 4H and 4I). IF staining of human GBM specimens showed that *ITGAL* (CD11a) protein was enriched in PKCδ^+^ macrophages, which exhibited significantly higher CD11a signals than their PKCδ^−^ counterparts (Figures 4J and 4K), thereby validating the transcriptomic differences observed between *Prkcd*^+^ and *Prkcd*^−^ microglia, monocytes, and MDMs.

### Protein kinase Cδ in microglia contributes to phagocytosis of glioblastoma cells: stimulation by niacin

**Figure 5:**
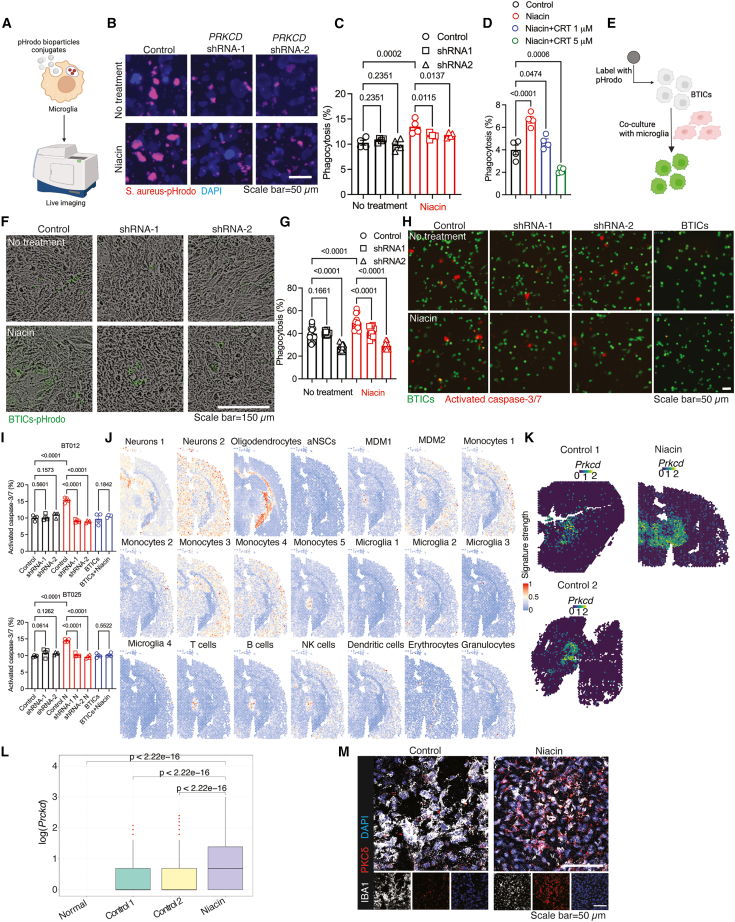
PKCδ in microglia contributes to the phagocytosis of BTICs (A) Schematic overview of the *in vitro* phagocytosis assay. (B and C) Representative IF images (B) and quantification (C) of phagocytosis of pHrodo-labeled *S. aureus* BioParticles by HMC3 microglia cells with *PRKCD* knockdown, in the presence or absence of niacin. (D) Phagocytosis assay using primary human microglia stimulated with niacin, with or without the PKC inhibitor CRT0066101. (E) Schematic of the *in vitro* phagocytosis assay using human BTICs labeled with pHrodo. (F and G) Representative images (F) and quantification (G) of phagocytosis of pHrodo-labeled human BTICs (BT012) by HMC3 cells with *PRKCD* knockdown. (H and I) Representative IF images (H) and quantification (I) of apoptotic BTICs (BT012 and BT025), determined by activated caspase-3/7 staining following co-culture with control or *PRKCD*-knockdown HMC3 cells, in the presence or absence of niacin. (J) Cell-type deconvolution of Visium spatial transcriptomics data from tumor-bearing mice treated with niacin. (K) Comparison of *Prkcd* expression between niacin-treated and control mice in spatial transcriptomics. (L) Quantification of *Prkcd* expression across spatial clusters. (M) IF staining of PKCδ and IBA1 in brain sections from vehicle- and niacin-treated mice. Statistical comparisons among multiple treatment groups were conducted using one-way ANOVA followed by Benjamini-Hochberg correction. Differences in *Prkcd* expression between spatial slides were assessed using the Wilcoxon rank-sum test (*p* < 0.05). Data in (C and D), and I are presented as mean ± SEM. Scale bars on IF images: 50 μm.

Because both mouse and human scRNA-seq datasets identify microglia as the principal source of *PRKCD* in GBM, we asked whether PKCδ controls microglia phagocytosis. We quantified particle uptake *in vitro* with pHrodo-labeled S. aureus, which fluoresces in acidified phagosomes (Figure 5A). Building on our earlier work showing that vitamin B3 (niacin) boosts macrophage phagocytosis,^14^^,^^28^ we first confirmed that niacin similarly enhances phagosome acidification in primary human microglia (Figures S13A and S13B) and therefore used it as the phagocytic stimulus. *PRKCD* in the human microglia line HMC3 was knocked down with two independent shRNAs delivered by lentivirus, and efficient protein depletion was verified (Figure S13C). Niacin significantly increased bioparticle uptake in control cells (Figures 5B, 4C, and S13C), but this response was significantly reduced after *PRKCD* silencing. Likewise, the pharmacological inhibition of PKCδ with CRT0066101^29^ blocked the niacin-induced increase in phagocytosis (Figure 5D), underscoring the essential role of PKCδ in this process.

To better mimic conditions resembling the TME, we utilized patient derived BTICs labeled with pHrodo and co-cultured them with HMC3 cells (Figure 5E). In the co-culture experiments, we used BTICs because of their critical role in tumor initiation and their higher resistance to therapy compared to differentiated tumor cells. We employed *in vitro* live imaging to monitor the phagocytosis of BTICs. In agreement with the findings from pHrodo S. aureus particles, the downregulation of *PRKCD* in human microglia significantly reduced the stimulatory effects of niacin on the phagocytosis of BTICs (Figures 5F and 5G). Because microglia activation should trigger cytotoxicity toward BTICs, we measured activated caspase-3/7—a marker of apoptosis—in two patient-derived BTIC lines cocultured with HMC3 microglia. Niacin-primed microglia significantly increased caspase-3/7 activation in both BTIC lines, whereas this apoptotic response was significantly reduced when *PRKCD* was silenced in the microglia (Figures 5H, 5I, and S13D).

To delineate the role of PKCδ in the TME, we applied spatial transcriptomics and scRNA-seq to brain tissues from niacin-treated and vehicle-treated GBM-bearing mice. InferCNV highlighted chromosomal aberrations that defined tumor regions, mirroring earlier analyses (Figures S14A–S14D). Niacin enhanced infiltration of the MG2 subset at the tumor margin (Figure 5J) and substantially elevated *Prkcd* expression in this zone (Figures 5K and 5L). Differential-expression analysis confirmed significant *Prkcd* up-regulation in spatial clusters 2 (tumor core) and 7 (peritumoral region) of niacin-treated brains (Figures S14E and S14F). IF for PKCδ and the macrophage marker IBA1 validated the transcriptomic data, revealing higher PKCδ levels in IBA1^+^ cells from niacin-treated mice than from controls (Figure 5M).

### *In vivo* overexpression of *Prkcd* in microglia via adeno-associated virus restrains intracranial tumor growth

**Figure 6:**
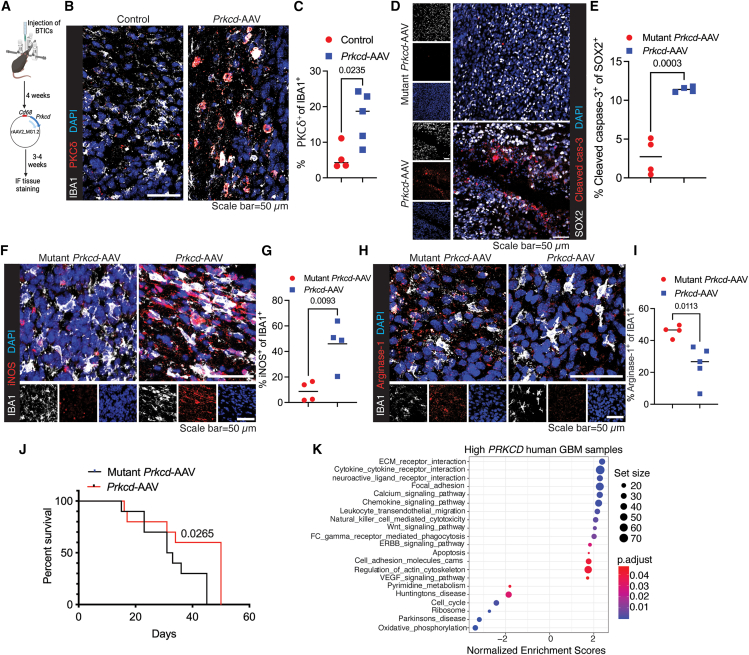
Overexpression of PKCδ in microglia via adeno-associated virus restrains intracranial tumor growth (A) Schematic of intratumoral injection of AAVs overexpressing *Prkcd* in microglia following tumor implantation. (B and C) Representative IF images (B) and quantification (C) of PKCδ expression in IBA1^+^ cells after *Prkcd*-AAV injection. (D and E) Representative IF images (D) and quantification (E) of cleaved caspase-3 expression in SOX2^+^ tumor cells from *Prkcd*-AAV-treated mice versus those treated with AAVs expressing a kinase-deficient *Prkcd* mutant. (F and G) Representative IF images (F) and quantification (G) of iNOS expression in IBA1^+^ cells. (H and I) Representative IF images (H) and quantification (I) of arginase-1 expression in IBA1^+^ cells. (J) Kaplan-Meier survival curves compare mice treated with *Prkcd*-AAVs or mutant *Prkcd*-AAVs. (K) Pathway enrichment analysis of TCGA human GBM samples stratified by high versus low *PRKCD* expression. Statistical significance in (C, E, G, and I) was determined using a two-tailed, unpaired *t* test. Survival differences in (J) were assessed using the log rank (Mantel-Cox) test. Differences in immune pathway enrichment (K) were evaluated using the Wilcoxon rank-sum test (*p* < 0.05). Data in (C, E, G, and I) are presented as mean ± SEM. Scale bars on IF images: 50 μm.

In a proof-of-concept study examining *in vivo* role of PKCδ in phagocytosis of BTICs and tumor control, we selectively overexpressed *Prkcd* in microglia using the microglia-tropic AAV-MG1.2 vector^30^ (Figure 6A). Tumor-bearing mice receiving AAV-*Prkcd* displayed significantly higher PKCδ protein in IBA1^+^ cells within the TME than untreated controls (Figures 6B and 6C). In a separate cohort, we compared AAV-*Prkcd* with an AAV encoding a kinase-dead *Prkcd* mutant (K376R, defective at the ATP-binding site).^31^ *Prkcd* overexpression significantly increased cleaved caspase-3-positive BTICs (Figures 6D and 6E), indicating that PKCδ^+^ microglia drive BTIC killing. Because PKCδ induces inducible nitric-oxide synthase (iNOS)^32^^,^^33^^,^^34^—a key mediator of phagocyte-mediated killing—we measured iNOS levels and found them significantly higher in macrophages from AAV-*Prkcd*-treated mice than in those receiving the kinase-deficient construct (Figures 6F and 6G). Finally, IF for arginase—a marker of immunosuppressive macrophages—showed that AAV-*Prkcd* significantly reduced the proportion of IBA1^+^/arginase^+^ cells (Figures 6H and 6I), indicating a shift toward an anti-tumor microglia phenotype. Survival analysis underscored the therapeutic advantage, showing that mice treated with AAV-*Prkcd* had significantly higher survival than those receiving the kinase-deficient mutant (Figure 6J).

We stratified TCGA GBM samples based on *PRKCD* expression and compared the transcriptomes of high- and low-expression groups. Tumors with high *PRKCD* levels exhibited elevated expression of genes related to ECM components (e.g., *COL8A1*, *COL5A1*, *COL1A2*, *LAMA3*) and immune/inflammatory signaling (*NLRP3*, *NFATC2*, *PRAM1*) (Figures S15A–S15B). Consistent with this expression profile, Immune, Stromal, and ESTIMATE scores were significantly higher in *PRKCD*-high tumors compared to *PRKCD*-low tumors (Figure S15C), suggesting increased accumulation of immune and stromal cells and reduced tumor purity in the *PRKCD*-high group. Pathway enrichment analysis further revealed upregulation of ECM-receptor interaction, cytokine-receptor interaction, and chemokine signaling pathways in *PRKCD*-high tumors (Figures 6K and S15D). Additionally, phagocytosis and apoptosis pathways were enriched, consistent with enhanced tumor-cell apoptosis potentially driven by PKCδ^+^ microglia. In contrast, *PRKCD*-low tumors showed negative enrichment for these immune-related and apoptotic pathways in IPA canonical pathway analysis (Figures S15D–S15F).

## Discussion

Our integrative single-cell and spatial transcriptomic analysis provides a comprehensive map of dynamic interactions between cells, including BTICs and microglia, within the GBM microenvironment. While previous studies have elucidated cellular heterogeneity within GBM and associated immune infiltrates using scRNA-seq and spatial methods,^6^^,^^20^^,^^21^^,^^35^ few have directly investigated the bidirectional crosstalk between BTICs and microglia at comparable resolution. Here, we not only characterize this intricate dialogue at single-cell and spatial scales but also identify and functionally validate a distinct PKCδ^+^ microglia subset exhibiting potent anti-BTIC activity.

The discovery of PKCδ-driven microglia reprogramming reveals an underexplored therapeutic avenue for targeting BTICs. Since microglia are resident immune cells that are present from the earliest stages of tumor development, reprogramming these cells offers a unique opportunity to intervene early and curb tumor growth at its origin. Utilizing two strategies—microglia-specific AAV-mediated *PRKCD* overexpression and systemic niacin administration—we demonstrate that PKCδ upregulation significantly enhances both phagocytosis of BTICs and induction of BTIC apoptosis, translating into survival benefits in a murine GBM model. Mechanistically, PKCδ promoted reactive oxygen species generation through gp91-phox (*CYBB*) and iNOS upregulation, mirroring antimicrobial phagocyte functions^36^^,^^37^ and highlighting a conserved ROS-mediated cytotoxic pathway. Notably, iNOS catalyzes the production of nitric oxide, which exerts cytotoxic effects on cancer cells.^38^^,^^39^ Critically, kinase activity was indispensable for these effects, as evidenced by the failure of the kinase-deficient K376R mutant to confer anti-tumor benefits, underscoring the necessity of downstream phosphorylation events.

Despite robust phenotypic changes of macrophages, survival analysis revealed only modest extensions in overall lifespan in our mouse GBM model. This outcome mirrors clinical observations in patients with GBM, where targeting a single molecule or mechanism often fails to overcome the inherent therapeutic resistance of the disease.^40^^,^^41^ The limited survival benefit observed in our study likely reflects the profound complexity of the GBM microenvironment, in which compensatory pathways, stromal interactions, and redundant immunosuppressive networks blunt the impact of single-target interventions. Effective elimination of BTIC-driven tumor growth and meaningful improvement in survival may therefore require combinatorial strategies that simultaneously engage multiple immune axes or incorporate PKCδ activation alongside standard-of-care therapies. Also, due to the significant heterogeneity of GBM cells, including BTICs, a recent study has shown that developing strategies targeting diverse cell populations of BTICs leads to better therapeutic outcomes.^18^

Although PKCδ expression in tumor cells has been reported to exhibit context-dependent roles,^42^^,^^43^^,^^44^ our data indicate that *PRKCD* transcripts in GBM are predominantly enriched in immune populations—particularly microglia, monocytes, MDMs, and dendritic cells—rather than malignant cells. The function of PKCδ in immune cells within cancer contexts remains poorly characterized. Recently, a study reported PKCδ expression in peripheral macrophages in animal models of breast cancer, lung cancer, and melanoma, demonstrating enhanced anti-tumor activity upon PKCδ deletion.^45^ These divergent outcomes likely reflect ontogenetic differences between yolk-sac-derived microglia and peripheral macrophages, as well as tumor-specific biology.

Future studies integrating our single-cell spatial atlas with functional genomic screens may uncover complementary targets that synergize with PKCδ activation. Additionally, combinatorial strategies pairing microglia reprogramming with immune checkpoint blockade or chemoradiation therapy merit further investigation to effectively counteract residual BTIC-driven recurrence. Our findings that niacin enhances PKCδ expression in human microglia informed the design of an ongoing phase 1/2a clinical trial evaluating niacin in patients with GBM (NCT04677049). In summary, our work reveals an underappreciated PKCδ-mediated axis of BTIC-microglia crosstalk, establishes proof-of-concept for the therapeutic reprogramming of resident brain microglia, and offers a detailed spatial-single-cell framework for developing next-generation GBM immunotherapies.

### Limitations of the study

Despite offering important insights into the GBM microenvironment and identifying PKCδ-expressing microglia with anti-tumor potential, our study has several limitations. First, while a key strength of this study is the targeting of BTICs, which are more resistant to therapy than other subsets of cancer cells, we did not evaluate the killing activity of PKCδ^+^ microglia against differentiated GBM cells. Second, although PKCδ upregulation enhanced microglia phagocytosis and killing of BTICs, the resulting survival benefit was modest, suggesting that PKCδ-based strategies alone may be insufficient and will likely need to be combined with other therapeutic modalities to achieve more robust and durable responses. Third, discriminating between resident microglia and infiltrating macrophages in the brain tumor microenvironment is challenging, particularly during inflammation when the expression of many markers’ changes. In this study, however, we used a newly developed AAV variant designed to specifically target microglia in the brain. The specificity of this approach, nevertheless, requires further validation. Addressing these limitations in future studies will be essential to fully evaluate and optimize the therapeutic potential of targeting PKCδ in GBM.

## Resource availability

### Lead contact

Further information and requests for resources and reagents should be directed to and will be fulfilled by the Lead Contact, V. Wee Yong (vyong@ucalgary.ca).

### Materials availability

This study did not generate new unique reagents.

### Data and code availability

scRNA-seq and spatial transcriptomics data have been deposited to the Gene Expression Omnibus (GEO) along with the appropriate metadata and are publicly available. The accession number is listed in the key resources table. All other data are available upon request. The analysis codes for the mouse scRNA-seq and spatial transcriptomics datasets presented in this study are available from the authors upon request.

## Acknowledgments

The authors gratefully acknowledge access to the Leica TCS SP8 and ImageXpress Micro XLS systems, as well as the accompanying image-analysis platforms, provided by the Hotchkiss Brain Institute Advanced Microscopy Platform (HBI-AMP) and the Cumming School of Medicine. We also thank the Cross Cancer Institute Imaging Facility (Dr. Xuejun Sun) for their support. Live-cell imaging was performed on the IncuCyte system at the Snyder Institute’s Live Cell Imaging Resource Laboratory, University of Calgary. Flow cytometry and DNA sequencing services were supplied by the University of Calgary Flow Cytometry Core and UC DNA Sequencing Facility, respectively. This work was funded by the 10.13039/501100000024Canadian Institutes of Health Research, the Canadian Cancer Society, 10.13039/501100000001The Alberta Cancer Foundation and Cancer Research Institute of Northern Alberta (CRINA). We are especially grateful to Dr. Chunhai Hao (Indiana University) for providing the human GBM specimens.

## Author contributions

R. Mirzaei: conceptualization, supervision, data curation, software, analysis, validation, visualization, methodology, writing – original draft, and writing – review and editing. R. McNeil: data curation, analysis, visualization, methodology, and writing – review and editing. B. Wong: data curation and writing – review and editing. C. D'Mello: software, analysis, single-cell gene expression and spatial library preparation, and writing – review and editing. S. Sarkar: methodology and writing – review and editing. F. Visser: methodology and writing – review and editing. C. Poon: writing – review and editing. P. Bose: supervision, software, and writing – review and editing. V.W. Yong: conceptualization, resources, supervision, funding acquisition, validation, project administration, and writing – review and editing.

## Declaration of interests

The authors decare no competing interests.

## Declaration of generative AI and AI-assisted technologies in the writing process

During the preparation of this work, the authors used [ChatGPT - OpenAI] to improve the readability and language of the article. After using this tool, the authors reviewed and edited the content as needed and take full responsibility for the content of the published article.

## STAR★Methods

### Key resources table

**Table:** 

REAGENT or RESOURCE	SOURCE	IDENTIFIER
**Antibodies**		

PKCδ	Abcam	Cat# ab182126; RRID: AB_2892154
IBA1	Thermo Fisher	Cat# PA5-18039; RRID:AB_10982846
Arginase (Arginase-1)	Cell Signaling	Cat# 93668; RRID:AB_2800207
Cleaved Caspase-3	Cell Signaling	Cat# 9661; RRID:AB_2341188
TMEM119	Abcam	Cat# ab209064; RRID:AB_2800343
CD68	BioLegend	Cat# 137001; RRID:AB_2044003
gp91-phox (CYBB)	Novus Biologicals	Cat# NBP1-41012; RRID:AB_3206985
CD11a (ITGAL)	Thermo Fisher	Cat# PA5-19161; RRID:AB_10978380
iNOS	Thermo Fisher	Cat# PA3-030A; RRID:AB_2152737
Alexa Fluor® 647-conjugated AffiniPure F(ab')2 Fragment Donkey Anti-Rat IgG (H+L) (min X Bov,Ck,Gt,GP,Sy Hms,Hrs,Hu,Ms,Rb,Shp Sr Prot)	Jackson ImmunoResearch Labs	Cat# 712-606-153; RRID:AB_2340696
Alexa Fluor® 488-AffiniPure F(ab')2 Fragment Donkey Anti-Mouse IgG (H+L) (min X Bov,Ck,Gt,GP,Sy Hms,Hrs,Hu,Rb,Rat,Shp Sr Prot)	Jackson ImmunoResearch Labs	Cat# 715-546-151; RRID:AB_2340850
Alexa Fluor® 647-conjugated AffiniPure F(ab')2 Fragment Donkey Anti-Goat IgG (H+L) (min X Ck,GP,Sy Hms,Hrs,Hu,Ms,Rb,Rat Sr Prot)	Jackson ImmunoResearch Labs	Cat# 705-606-147; RRID:AB_2340438
Alexa Fluor® 488-conjugated AffiniPure F(ab')2 Fragment Donkey Anti-Mouse IgG (H+L) (min X Bov,Ck,Gt,GP,Sy Hms,Hrs,Hu,Rb,Shp Sr Prot)	Jackson ImmunoResearch Labs	Cat# 715-546-150; RRID:AB_2340849
Alexa Fluor® 647-conjugated AffiniPure F(ab')2 Fragment Donkey Anti-Mouse IgG (H+L) (min X Bov,Ck,Gt,GP,Sy Hms,Hrs,Hu,Rb,Shp Sr Prot)	Jackson ImmunoResearch Labs	Cat# 715-606-150; RRID:AB_2340865
Alexa Fluor® 488-conjugated AffiniPure F(ab')2 Fragment Donkey Anti-Rat IgG (H+L) (min X Bov,Ck,Gt,GP,Sy Hms,Hrs,Hu,Ms,Rb,Shp Sr Prot)	Jackson ImmunoResearch Labs	Cat# 712-546-153; RRID:AB_2340686
Alexa Fluor® 488-conjugated AffiniPure F(ab')2 Fragment Donkey Anti-Goat IgG (H+L) (min X Ck,GP,Sy Hms,Hrs,Hu,Ms,Rb,Rat Sr Prot)	Jackson ImmunoResearch Labs	Cat# 705-546-147; RRID:AB_2340430
Goat anti-Rabbit IgG (H+L) Cross-Adsorbed Secondary Antibody, Alexa Fluor 546	Thermo Fisher	Cat# A-11010; RRID:AB_2534077

**Biological samples**		

Human GBM tissue samples	Indiana University	NA

**Chemicals, peptides, and recombinant proteins**		

Epidermal Growth Factor (EGF)	STEMCELL Technologies	Cat# 78006.2
Fibroblast Growth Factor (FGF)	STEMCELL Technologies	Cat# 78003.2
Niacin (Vitamin B3)	Sigma	Cat# N4126
Pluronic® F-68	Sigma	Cat# P1300
PEIpro	Polyplus	Cat# 101000017
CRT0066101	Tocris	Cat# 49753
Puromycin dihydrochloride from Streptomyces	Sigma	Cat# P7255
Polybrene	Sigma	Cat# TR-1003-G
NeuroCult™ Basal Medium (Mouse & Rat)	STEMCELL Technologies	Cat# 05700
MEM	Thermo Fisher	Cat# 11095080
Glutamax™ Supplement	Thermo Fisher	Cat# 35050061
Sodium Pyruvate (100 mM)	Thermo Fisher	Cat# 11360070
Penicillin Streptomycin	Thermo Fisher	Cat# 15140122
Fetal Bovine Serum	Corning	Cat# 35-077-CV
Heparin Solution	STEMCELL Technologies	Cat# 07980

**Critical commercial assays**		

pHrodo™ S. aureus BioParticles	Thermo Fisher	Cat# P3536
pHrodo™ Red SE (Succinimidyl Ester)	Thermo Fisher	Cat# CP36600
NucBlue™ Live ReadyProbes™ Reagent	Thermo Fisher	Cat# R37605
CellTracker™ Green CMFDA Dye	Thermo Fisher	Cat# C7025
CellEvent™ Caspase-3/7 Red Detection Reagent	Thermo Fisher	Cat# C10430
SYTOX™ Red Dead Cell Stain	Thermo Fisher	Cat# S34859
Venor™ GeM Mycoplasma Detection Kit	Sigma	Cat# MP0025
Visium Spatial Gene Expression Slide & Reagent kit	*10x Genomics*	V10N30-034
Visium Spatial Tissue Optimization Slide & Reagent kit	*10x Genomics*	201012

**Deposited data**		

Single-cell RNA-seq of mouse GBM	This study	NCBI GEO: GSE298688
Single-cell RNA-seq of human GBM	Richards et al.^5^	EGA: EGAS00001004656
Spatial transcriptomics mouse GBM (niacin group)	This study	NCBI GEO: GSE298689
Spatial transcriptomics mouse GBM	Mirzaei et al.	NCBI BioProject: PRJNA914489

**Experimental models: Cell lines**		

HMC3 – Human microglial cell line	ATCC	CRL-3304
BT0309 – Mouse brain tumor–initiating cell (BTIC) line	Derived from NPcis (Nf1+/–;Trp53+/–) C57BL/6J mice	University of Calgary
BT048	Derived from GBM patients	University of Calgary BTIC Core
BT012	Derived from GBM patients	University of Calgary BTIC Core
BT025	Derived from GBM patients	University of Calgary BTIC Core
BT073	Derived from GBM patients	University of Calgary BTIC Core

**Experimental models: Organisms/strains**		

Mouse: C57BL/6NCrl	Charles River Laboratories	027; RRID:IMSR_CRL:027

**Recombinant DNA**		

Plasmid: pHAGE-PGK-GFP-IRES-LUC-W	Addgene	46793
Plasmid: psPAX2	Addgene	12260
Plasmid: pMD2.G	Addgene	12259
Plasmid: pAAV-CD68-hM4D(Gi)-mCherry	Addgene	75033
Plasmid: Rep-Cap plasmid encoding MG1.2 capsid	Addgene	184541

**Software and algorithms**		

FlowJo	BD	https://www.flowjo.com
GraphPad Prism (v10.1.0)	Graphpad	https://graphpad.com
Fiji (ImageJ)	Schindelin et al.	https://imagej.net/software/fiji/
R 4.0.4	The R Foundation for Statistical Computing	https://www.r-project.org/
BioRender	BioRender.com	https://www.biorender.com
IMARIS	Oxford Instruments	https://imaris.oxinst.com/
Qiagen Ingenuity Pathway Analysis (IPA)	*QIAGEN*	https://digitalinsights.qiagen.com/products-overview/discovery-insights-portfolio/analysis-and-visualization/qiagen-ipa
Leica Application Suite X (LAS X)	Leica	https://www.leica-microsystems.com/products/microscope-software/p/leica-las-x-ls/
Spatial Ranger 2.1.0	10x Genomics	https://10xgenomics.com/
Seurat 3.1.1 (R 4.0.0)	Butler et al.	https://satijalab.org/seurat/articles/get_started.html
pheatmap 1.0.12 (R 4.0.0)	Kolde et al.	https://cran.r-project.org/web/packages/pheatmap/
ggplot2 3.4.2 (R 4.0.0)	Wickham et al.	https://cran.r-project.org/web/packages/ggplot2/index.html
CellChat 1.6.1 (R 4.0.0)	Jin et al.	https://github.com/sqjin/CellChat
Loupe Browser 6	10× Genomics	https://www.10xgenomics.com/support/software/loupe-browser/latest
InferCNV, version 1.2.2	Tickle et al., 2019	https://www.bioconductor.org/packages/infercnv/
Monocle v3	Trapnell et al., 2014 and Qiu et al., 2017	https://bioconductor.org/packages/monocle/
CARD	Ma et al.	https://github.com/YMa-lab/CARD
DESeq2	Bioconductor	https://bioconductor.org/packages/DESeq2
ClusterProfiler	Bioconductor	https://bioconductor.org/packages/clusterProfiler
Harmony	Korsunsky et al.	https://github.com/immunogenomics/harmony?tab=readme-ov-file
SingleR	Bioconductor	https://bioconductor.org/packages/SingleR
MetaXpress	Molecular Devices	https://www.moleculardevices.com
IncuCyte Live-Cell Analysis Software	Sartorius	https://www.sartorius.com

### Experimental model and study participant details

#### Mice

All *in vivo* experiments were performed using 6–8-week-old female C57BL/6 wild-type mice obtained from Charles River Laboratories. Only females were used to minimize variability associated with sex-specific differences in tumor growth and immune responses; however, this represents a limitation because sex-based effects cannot be directly evaluated. Mice were housed in a pathogen-free facility under a 12-h light/dark cycle with *ad libitum* access to food and water. All procedures were approved by the University of Calgary Animal Care Committee and were conducted in accordance with Canadian Council on Animal Care (CCAC) guidelines. Mice were randomly assigned to experimental groups, and sample sizes were chosen based on prior studies.

#### Primary human microglia

Human fetal microglia (18–22 weeks gestation) were isolated from mixed brain-tissue preparations using a published protocol.^46^ All tissue procurement and experimental procedures were approved by the University of Calgary Research Ethics Board, with informed consent obtained in accordance with institutional and national guidelines. Microglia were cultured in MEM supplemented with L-glutamine, sodium pyruvate, 10 mM dextrose, 1% penicillin–streptomycin, and 10% fetal bovine serum (Invitrogen). The sex of fetal donors was not available, representing a limitation for interpreting potential sex-dependent effects.

#### Human microglia cell line

The human microglia cell line HMC3 (ATCC CRL-3304) was used for in-vitro validation experiments, including *PRKCD* knock-down assays. HMC3 cells were maintained in the same MEM-based medium and supplements described for the primary human microglia.

#### Human GBM tissues

Human glioblastoma (GBM) surgical specimens (n = 8) were collected under Indiana University IRB–approved protocols, with written informed consent obtained from all participants. Fresh tissues were snap-frozen at −80 °C and later formalin-fixed and paraffin-embedded (FFPE) for histological and immunofluorescence analyses. Hematoxylin-and-eosin slides were reviewed by a board-certified neuropathologist. Patient demographic and clinical characteristics have been reported previously.^20^^,^^47^ The sex of the patients was available for all samples; however, given the limited cohort size, formal sex-stratified analyses could not be performed.

#### GBM patient derived BTICs

Patient-derived BTIC lines were generated from freshly resected GBM tissues following established protocols.^14^^,^^17^^,^^48^ Lines were expanded, cryopreserved, and authenticated within the University of Calgary BTIC Core Facility. For this study, we used four previously characterized BTIC lines (BT012, BT025, BT048, BT073).^20^^,^^47^ Cell identity was verified by STR profiling, and cultures were screened regularly for Mycoplasma contamination using the Venor GeM kit. The sex of the patient donors for each BTIC line was recorded; however, due to limited sample size, sex-based comparisons were not statistically powered and are therefore acknowledged as a limitation.

#### Mouse BTIC cell line

Mouse BT0309 cell line was originally generated from C57BL/6J NPcis *Trp53*^*+/-*^/*Nf1*^*+/-*^ mice and previously characterized.^24^ This cell line was cultured in stem cell enriched media supplemented with EGF (20 ng/mL) and FGF (20 ng/mL) and heparin solution (2 μg/mL) (Stem Cell Technologies).

### Method details

#### Live cell imaging of *in vitro* phagocytosis

Microglia were seeded into black-walled 96-well plates (20,000–30,000 cells/well) in complete medium and allowed to attach for 24 h at 37 °C, 5% CO_2_. The medium was then replaced with DMEM +1% FBS. After a 1-h equilibration, niacin (100 μM) was added, and cells were incubated for a further 12 h under the same conditions. The medium was aspirated and pHrodo™ S. aureus BioParticles (Thermo Fisher, P35361) or pHrodo™ Red–labelled BTICs (succinimidyl ester; Thermo Fisher, P36600) were added in live-cell imaging solution (10,000 particles or cells well^-1^). Nuclei were counter-stained with NucBlue Live ReadyProbes (2 drops mL^-1^; Thermo Fisher, R37605). Phagocytosis was imaged for 1 h on an ImageXpress Micro system (Molecular Devices) and continuously monitored thereafter on an Incucyte live-cell imager. The percentage of NucBlue-positive microglia that became pHrodo-positive was quantified with MetaXpress or Incucyte software.

#### Live cell imaging of *in vitro* caspase 3/7 assay

HMC3 microglia cells were plated at 20,000–30,000 cells per well in black-walled 96-well plates. After an overnight attachment, the medium was replaced with MEM supplemented with 1 % FBS. Cells were equilibrated for 1 h and then treated with niacin (Sigma, N4126; 100 μM) for 12 h at 37 °C, 5 % CO_2_. Patient-derived BTICs were dissociated to single cells and labelled with CellTracker™ Green CMFDA (1 μM; Thermo Fisher, C7025). Labelled BTICs (10,000 cells well^-1^) were added to the HMC3 monolayer and co-cultured overnight. CellEvent Caspase-3/7 Red Detection Reagent (Thermo Fisher, C10430) was then added, and plates were imaged 30 min later on an ImageXpress Micro Confocal High-Content system (Molecular Devices). The proportion of BTICs positive for Caspase-3/7 Red was quantified with MetaXpress software and expressed as the percentage of total CellTracker-positive cells.

#### Intracranial tumor implantation

After dissociation of mouse BTIC spheres, 25,000–50,000 viable cells from either firefly luciferase–GFP–expressing BTIC0309 were resuspended in 2 μL of PBS and stereotactically implanted into the right striatum of mice, as previously described.^49^ In the niacin experiments, starting seven days after implantation, mice were treated with niacin at a dose of 100 mg/kg daily via intraperitoneal injection until the termination of the experiment. Control mice were injected with an identical vehicle (saline) solution.

#### Preparation of libraries for spatial transcriptomics

The spatial transcriptomics analysis was conducted using the Visium Spatial Gene Expression platform by 10x Genomics. Brain tissues, collected 40 days after tumor implantation, were frozen and sectioned into 10-μm slices, following the manufacturer’s guidelines. Sections were placed on Visium slides and processed as per the user guide. Tissue optimization studies delineated an optimal permeabilization time of 12 mins. Brightfield images distinguishing tumor and non-tumor regions were captured using the EVOS FL Auto Imaging System. The subsequent library preparation steps were performed as per the user guide (Visium Spatial Gene Expression Reagent Kit; V10N30-034). The libraries were then sequenced on an Illumina NovaSeq 6000 system, employing a NovaSeq 200 Cycle S1 flow cell and specific read cycles for sequencing.

#### Confocal immunofluorescence microscopy

We analyzed samples from GBM patients fixed in formalin and embedded in paraffin, as well as frozen mouse sections, following established protocols.^20^^,^^47^ Following antibodies were used for tissue staining: PKCδ (Abcam, ab182126, 1:200 dilution), IBA1 (Thermo Fisher, PA5-18039, 1:100 dilution), Arginase (Cell Signaling, 93668, 1:50), Cleaved Caspase-3 (Cell Signaling, 9661, 1:500), TMEM119 (Abcam, ab209064, 1:400 dilution), CD68 (Biolegend, 137001, 1:500), gp91-phox (Novus Biologicals, NBP1-41012, 1:100), CD11a (Thermos Fisher, PA5-19161, 1:100) and iNOS (Thermo Fisher, PA3-030A, 1:500). After primary antibody incubation, samples were treated with corresponding fluorophore-conjugated secondary antibodies (1:500; Jackson ImmunoResearch Laboratories or Thermo Fisher Scientific) and 4′,6-diamidino-2-phenylindole (DAPI; 1:1000). Laser confocal IF imaging was performed at room temperature using the Leica TCS SP8 microscope with consistent settings across samples, ensuring optimal contrast and minimal saturation. Each sample generated three to four fields of view (FOV). Image acquisition was done using Leica Application Suite X, and subsequent picture thresholding and three-dimensional (3D) rendering were conducted with Imaris software (v9.9.1 Bitplane).

#### Lentivirus production

Lentiviral vectors carrying shRNAs against human *PRKCD* (NM_006254) or a non-target control were purchased from Sigma (TRCN0000010193, TRCN0000195408, SHC002). For GFP^+^ luciferase co-expression in mouse BT0309 cells we used pHAGE-PGK-GFP-IRES-LUC-W (Addgene #46793; gift of D. Kotton). For virus production, 293FT cells were grown to ∼90% confluence in five 15-cm dishes and co-transfected with 112.5 μg transfer vector, 73 μg psPAX2 and 39.5 μg pMD2.G (Addgene #12260; gifts of D. Trono) using PEIpro (Polyplus). After 48 h, supernatants were collected, clarified (500 g, 5 min), passed through a 0.45 μm filter, under-laid with 10 % sucrose and ultracentrifuged (12,000 g, 4 h, 4 °C).^26^ Pellets were resuspended in sterile PBS and stored at –80 °C in 20 μL aliquots. Titres measured by qPCR (Applied Biological Materials) were > 1 × 10^9^ IU mL^-1^. For transduction, HMC3 or BT0309 cells were seeded at 2 × 10^5^ cells well^-1^ in 6-well plates; the following day, concentrated virus was added with polybrene (1 μg mL^-1^).

#### Plasmid construction for *in vivo* expression of PKCδ

pAAV-CD68-hM4D(Gi)-mCherry, with the monocyte-specific promoter CD68 and was a gift from B. Roth (Addgene plasmid no. 75033^50^), was digested with SalI and HindIII to excise the hMD(Gi)-mCherry sequence. The cDNA sequence corresponding to mouse *Prkcd* (accession AF251036) was amplified from a mouse brain cDNA library and subcloned to generate pAAV-CD68-*Prkcd* or pAAV-CD68-*Prkcd*-KR (K376R mutant with ATP binding deficiency^31^) using the NEBuilder Hifi DNA assembly kit. All DNA constructs were verified by Sanger DNA sequencing.

#### AAV production for *in vivo* overexpression of PKCδ in microglia and macrophages

AAV vectors bearing the MG1.2 capsid were produced as previously described.^51^ Briefly, 293FT cells were grown to ∼90 % confluence in Corning HyperFlasks and co-transfected with 129 μg pHELPER (Agilent), 238 μg Rep-Cap plasmid encoding MG1.2 (Addgene #184541, gift of M. Luo) and 64.6 μg pAAV-*CD68*-*Prkcd* or pAAV-*CD68*-*Prkcd*-KR using PEIpro (Polyplus). Supernatants were collected at days 3 and 5, mixed with cells harvested on day 5, and virus was precipitated with 40 % PEG/2.5 M NaCl. The pellet was resuspended in 500 mM NaCl, 40 mM Tris, 10 mM MgCl_2_ and treated with salt-active nuclease (100 U mL^-1^, 37 °C, 1 h). After clarification (2,000 g, 15 min), AAVs were purified on a 15/25/40/60 % iodixanol step gradient (OptiSeal tubes; 350,000 g, Type 70 Ti rotor). The 40 % fraction was withdrawn, diluted in PBS + 0.001 % Pluronic F-68, and passed through a 0.2 μm filter. Virus was concentrated and buffer-exchanged by five spins in Amicon Ultra-15 filters (100 kDa cutoff). Genome copy titre was determined with the ABM qPCR AAV Titration Kit; purity was confirmed by SDS–PAGE with InstantBlue total-protein stain (Expedeon).

#### *In vivo* overexpression of PKCδ in myeloid cells with AAVs

To overexpress mouse PKCδ (*Prkcd*) in the brain of tumor-bearing mice, a solution of 5 μl saline containing 2.5 × 10ˆ10 genome copies of AAV-MG1.2, designed with MG-targeting potential, was intratumorally injected. The AAV-MG1.2 construct was engineered to overexpress *Prkcd* under the control of the CD68 promoter. This procedure was performed 2 weeks after tumor injection. Control mice were injected with 5 μl of saline containing 2.5 × 10ˆ10 genome copies of AAV-MG1.2 with a CD68 promoter designed to express a mutant version of *Prkcd* (*Prkcd*-KR).

#### Single cell isolation for scRNA-seq

Mice were euthanized four weeks post-tumor implantation, and brains were isolated. To enrich for tumor tissue, brains were hemisected, and a 2 mm section around the tumor injection site was collected into Hank’s Balanced Salt Solution (HBSS, Gibco). Brain tissues were pooled together per sample to ensure an adequate number of cells for downstream scRNA-seq analysis (Control: 5 mice, one replicates; niacin injection: 10 mice, two replicates). Subsequently, brain tissues were homogenized using a Dounce homogenizer and filtered through a 40-μm cell strainer. The cell suspension underwent Percol gradient separation to remove myelin debris and enrich leukocytes. After further filtration through a 35-μm cell strainer, cells were resuspended in PBS containing 1% heat-inactivated FBS (Sigma) and 0.1 mM EDTA.

#### scRNA-seq sample and library preparation

Single-cell suspensions were stained with SYTOX Red Dead Cell Stain (Thermo Fisher Scientific, S34859) to exclude non-viable cells, and live cells were sorted on a FACSAria III (BD) equipped with a 100 μm nozzle. Gene-expression libraries were generated with Chromium Next GEM Single Cell 3′ v3.1 kits (10x Genomics). A cell volume calculated to recover ∼3,000 cells (per the manufacturer’s guide) was loaded onto a Chromium controller chip. cDNA was amplified, quality-checked, and quantified with a High-Sensitivity DNA chip on an Agilent 2100 Bioanalyzer; the same platform was used for final library QC. Libraries were pooled and sequenced on an Illumina NovaSeq 6000 (S1 flow cell) at 300 pM. Read 1 (28 bp) captured cell barcodes and UMIs, the i7 index read (8 bp) recorded sample indices, and Read 2 (91 bp) sequenced transcript inserts, using paired-end mode.

#### Single-cell RNAseq data processing

Sequencing generated ∼95,000–116,000 reads per cell. BCL files were processed with Cell Ranger v3.1: cellranger count used STAR to align reads to the mm10 reference, and cellranger aggregate merged libraries without additional normalization. In Seurat, cells with fewer than 200 detected genes or more than 20 % mitochondrial reads were discarded, yielding 5,440 high-quality cells for analysis. After normalization and scaling, cells were clustered at a resolution of 0.5. Cluster-specific markers were identified with Seurat’s FindAllMarkers function.

#### Cell type deconvolution of scRNA-seq data

Single-cell RNA-seq deconvolution was performed with SingleR and CellDex, using a mouse bulk RNA-seq reference from CellDex. Cell-type assignments from SingleR were cross-checked against canonical marker genes. Because several biological cell types spanned multiple Seurat clusters, we carried out a second, higher-resolution analysis that combined Seurat clustering, differential-expression profiles, pseudotime trajectories (Monocle3), and pathway-activity inference (GSEA/MSigDB Hallmarks, GO, KEGG, and Reactome via ClusterProfiler). This approach resolved five monocyte subsets: Group 1 (monocytes 1 and 3; pseudotime point 2), Group 2 (monocytes 2 and 4; point 3), and Group 3 (monocyte 5; point 1). Two macrophage populations (MDM1 and MDM2) were detected but did not subdivide further upon reclustering. Four distinct microglia subtypes (MG1–MG4) emerged; MG1 (point 2) and MG2 (point 3) were transcriptionally related, whereas MG3 and MG4 (both point 1) formed separate branches.

#### CellChat – Cell to cell interaction analysis

The mouse GBM scRNA-seq dataset was used as input for a CellChat analysis, and cell type identities were set to SingleR deconvolution/ reclustering analysis results. The mouse proportion of the cell-to-cell interaction database was used, along with secreted ligand, ECM receptors, and cell-cell contact databases. CellChat identified differentially expressed ligand-receptor genes between cell types, and identified significant pathways related to cell-to-cell interactions. The basic CellChat pipeline was used to identify interactions between cell types and incoming/ outgoing interactions was determined using non-negative matrix factorization (NMF).

#### Qiagen Ingenuity Pathway Analysis of single cell and TCGA data

Differential expression results from scRNA-seq and TCGA analysis were used to run an IPA core analysis and examine canonical pathway and upstream regulator activity between cell types (scRNA-seq) and expression groups (*PRKCD* High vs Low – TCGA data). Differential expression results from Seurat FindAllMarkers were used as input for IPA using previously defined log2 foldchange and p-adjusted value for scRNA-seq data. Two differential expression analysis were performed, one with DEGs in high *PRKCD* expression samples, and DEGs in low *PRKCD* expression samples in TCGA data. Two separate IPA core analysis were run on the two separate gene sets for TCGA. IPA results were downloaded and visualized using the ggplot2 package.

#### Pathway analysis using ClusterProfiler R package to perform GSEA of MSigDB pathways

Secondary pathway analysis was performed using the Clusterprofiler R package, to perform geneset enrichment analysis (GSEA) to identify enriched geneset/ pathways from the Mutational Signatures Database (MSigDB). This database contains gene sets from various sources and for this analysis included the hallmarks of cancer gene sets, KEGG, REACTOME, and Gene Ontology (GO) pathways. The pathway analysis was used DEGs from DEAs to identify the enrichment of pathway activity across scRNA-seq and bulk RNA-seq data. This method was used to identify pathway activity in immune cell types in mouse and human scRNA-seq data using DEGs of each cell type. This method was also used for TCGA GBM RNA data for DEGs from *PRKCD* high vs low samples.

#### Data processing and batch correction of spatial transcriptomics

Spatial transcriptomics data was imported (images, counts, scale factors, and tissue position files) using Seurat and the CleanSpot R packages. Four tissue slides/samples were merged containing one normal tissue slide with no tumor, one tissue slide with tumor and niacin treatment, and two tissue slides with tumor and no niacin treatment. Initial data processing removed lowly expressed genes and spots with low feature counts. The merged spatial Seurat object was normalized (SCTransform, PCA components = 30), scaled, and then spots were clustered (res = 0.5) as per basic Seurat processing protocols. Next, spatial slides were checked for sample-based batch effects, and then these effects were removed using the Harmony R package. After batch correction, spots clustered based on biological gene expression and not by tissue slide/ samples. After initial data processing there were 12,588 spots ready for downstream analysis. After default Seurat clustering, differential expression analysis was performed between Seurat clusters using the FindAllMarkers function, thresholds for DEGs were set to log2fold change +/- 0.58 and p-adjusted value < 0.05.

#### Spot deconvolution in spatial transcriptomics

Spatial deconvolution was performed with the CARD R package to assign cell type identity to spots by integrating our in-house mouse GBM single-cell data as a reference. CARD uses autoregressive-based deconvolution to combine cell type specific expression information from scRNA-seq data with correlation in cell type composition across tissue locations. Each spot on the tissue slide was assigned a proportionality score of multiple cell types detected within each spot. To validate and confirm CARD results we also performed deconvolution using the SingleR to identify concordant calls between the two methods.

#### InferCNV – Cancer/tumor spot identification

CNV events were used to identify tumor cells within spots of spatial slides. The InferCNV pipeline was run as per default, but importantly noting we used the Seurat clusters designated as “cancer spots – cluster 2” and “normal spots – all other clusters” to compare and identify CNV events in cancer vs normal spots. Tumor cells/spots were identified using the top duplication event identified, tumor spot identification overlapped nicely with visual images of tumor location on the tissue slide. Differential expression analysis was performed between identified cancer/ tumor spots and adjacent normal spots to validate InferCNV calls by identifying cancer-associated upregulated genes within cancer/ tumor spots.

#### Bulk RNA-seq TCGA GBM data download and processing

GBM mRNA-seq data (expected counts and TPM-normalised values) were downloaded from the TCGA hub on UCSC Xena. Expected counts were analysed with DESeq2, while TPM values served all other downstream applications, including immune-cell deconvolution. For the *PRKCD*-centred differential-expression analysis, tumours were stratified into *PRKCD* quartiles, with quartile 1 representing low and quartile 4 high expression. Samples from quartiles 1 and 4 were compared in DESeq2 (default settings). Genes were deemed differentially expressed at |log_2_FC| > 0.58 with an adjusted P < 0.05, and results were visualised as volcano plots and heat-maps using ggplot2 and pheatmap.

#### Analysis of public scRNA-seq datasets

The raw counts matrix from Richards et al.^5^ was imported into Seurat v3 (R) and converted to a Seurat object. Quality filtering removed cells with < 50 detected genes or > 15 % mitochondrial transcripts. Data were then integrated and normalized with SCTransform. Dimensionality reduction used principal-component analysis (15 significant PCs), followed by graph-based clustering (FindNeighbors, FindClusters, resolution = 0.5). Clusters were manually annotated according to lineage-specific marker expression. Visualization employed RunTSNE (PCA input). Differentially expressed genes (cluster vs. all others) were identified with FindMarkers; genes with adjusted P < 0.05 were retained. Heat-maps (DoHeatmap), dot plots (DotPlot), feature plots (FeaturePlot) and violin plots (VlnPlot) were generated to display DEG patterns and hallmark-gene expression across clusters.

#### Immune contexture analysis

The immune cell contexture of GBM high *PRKCD* verses *PRKCD* low expression of TCGA samples were examined using two methods. Firstly, the ESTIMATE algorithm was used to examine immune scores indicating overall levels of immune cell infiltration between the two expression groups, which also included ESTIMATE stromal and purity scores. Secondly, a paper published using immune cell markers validated by flow cytometry, immunohistochemistry, and know cell type marker databases was used to deconvolute and assign tumor-infiltrating immune (TIL) scores per TCGA sample including GBM samples. *PRKCD* expression groups (high = 4 vs low = 1) were compared for differences in tumor-infiltrating immune scores using Wilcoxon T-test (p-adj value < 0.05). The TIL score dataset came from Danaher et al.^52^

### Quantification and statistical analysis

#### Confocal image analysis

Z-stack files were opened in Imaris in their native format. Marker-positive cells were segmented with the Surface tool after background subtraction. A uniform fluorescence-intensity threshold—calibrated on sections stained with secondary antibody only—was applied to all images within an experiment, together with fixed size and circularity filters, to exclude nonspecific labeling. Double-positive cells were counted via the “shortest distance between surfaces” metric. Quantitative outputs were exported to Excel for downstream analysis. Representative images were generated in ImageJ as maximum-intensity projections of each channel, displayed with pseudocolors. Brightness and contrast (and, when illustrating, equal signal boosting) were adjusted identically across samples to enhance clarity without bias.

#### Statistical methodology

Data were organized in Microsoft Excel and plotted in GraphPad Prism (v10.1.0). Each graph displays individual data points—one point per mouse for *in vivo* studies or per well in a 96-well plate for *in vitro* assays—overlaid with the mean ± SEM. Sample sizes were based on prior publications,^19^^,^^53^ while balancing cost, feasibility, and the availability of sex- and age-matched mice. Mice were randomly allocated to experimental groups and treatments. Statistical significance was assessed with one-way ANOVA followed by the Benjamini–Hochberg multiple-comparison test when comparing two or more treatment groups with the control, and with a two-tailed unpaired t-test for comparisons involving only two groups. Experiments were not blinded.
