# Supplementary material for: Spatial single-cell profiling identifies protein kinase Cδ-expressing microglia with anti-tumor function in glioblastoma
Source: iScience. 2025 Nov 29;29(1):114281. doi: 10.1016/j.isci.2025.114281 (PMC12768871; doi:10.1016/j.isci.2025.114281)
Supplement: Document S1. Figures S1–S15 [file mmc1.pdf]

iScience, Volume *29*

Supplemental information

Spatial single-cell profiling identifies protein kinase Cδ-expressing microglia with anti-tumor function in glioblastoma

Reza Mirzaei, Reid McNeil, Charlotte D'Mello, Britney Wong, Susobhan Sarkar, Frank Visser, Candice Poon, Pinaki Bose, and V. Wee Yong

Figure S1

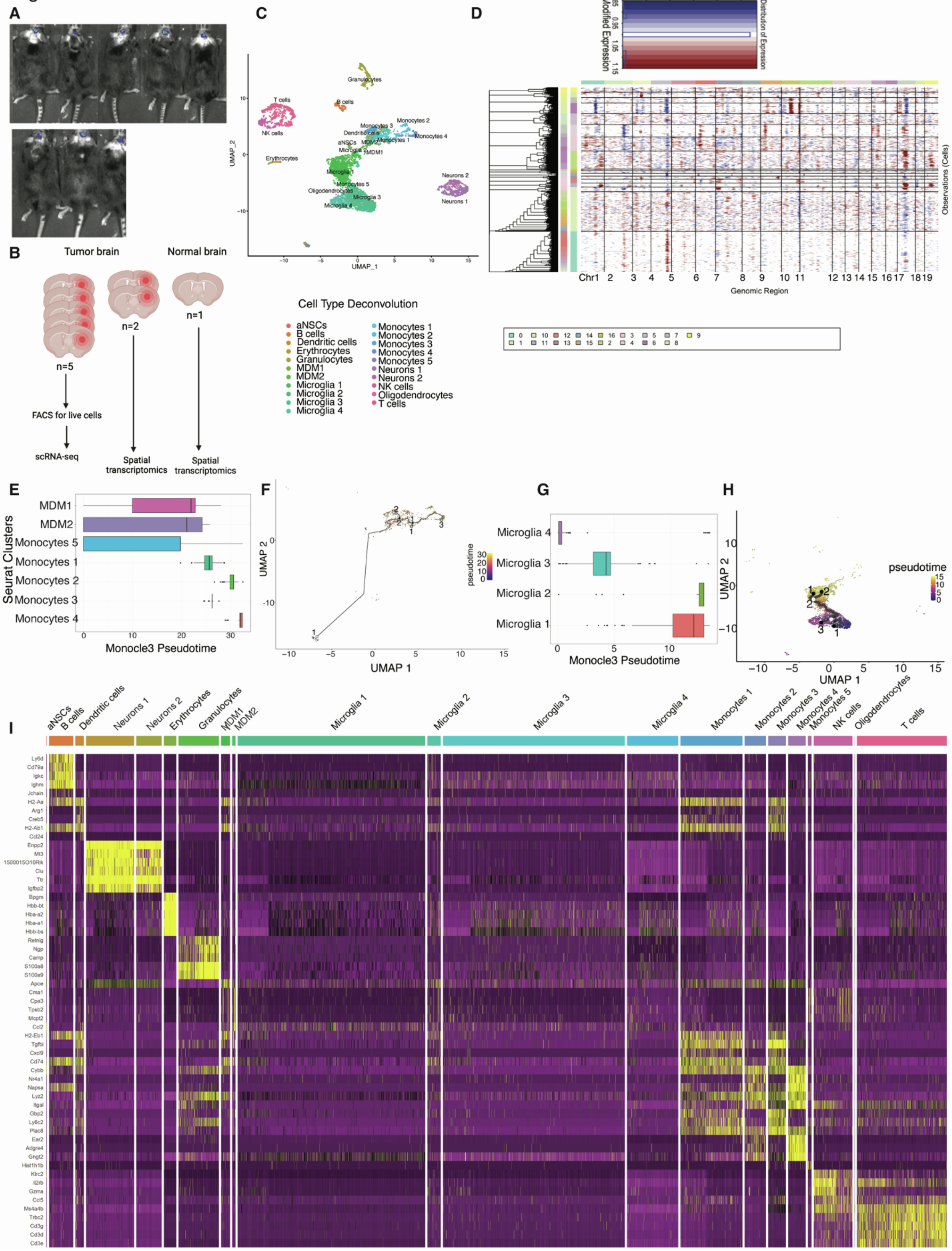

A
B
Tumor brain
Normal brain
n=5
n=2
n=1
FACS for live cells
scRNA-seq
Spatial transcriptomics
Spatial transcriptomics
C
UMAP_1
UMAP_2
Granulocytes
T cells
B cells
NK cells
Erythrocytes
Monocytes 2
Monocytes 3
Dendritic cells
Monocytes 4
Monocytes 1
aNSCs
MDM2
MDM1
Microglia 2
Microglia 1
Monocytes 5
Oligodendrocytes
Microglia 3
Microglia 4
Neurons 2
Neurons 1
Cell Type Deconvolution
aNSCs
B cells
Dendritic cells
Erythrocytes
Granulocytes
MDM1
MDM2
Microglia 1
Microglia 2
Microglia 3
Microglia 4
Monocytes 1
Monocytes 2
Monocytes 3
Monocytes 4
Monocytes 5
Neurons 1
Neurons 2
NK cells
Oligodendrocytes
T cells
D
Count
Modified Expression
Distribution of Expression
Observations (Cells)
Chr1 2 3 4 5 6 7 8 9 10 11 12 13 14 15 16 17 18 19
Genomic Region
E
MDM1
MDM2
Monocytes 5
Monocytes 1
Monocytes 2
Monocytes 3
Monocytes 4
Seurat Clusters
Monocle3 Pseudotime
F
UMAP 1
UMAP 2
G
Microglia 4
Microglia 3
Microglia 2
Microglia 1
pseudotime
Monocle3 Pseudotime
H
pseudotime
UMAP 1
UMAP 2
I
aNSCs
B cells
Dendritic cells
Neurons 1
Neurons 2
Erythrocytes
Granulocytes
MDM1
MDM2
Microglia 1
Microglia 2
Microglia 3
Microglia 4
Monocytes 1
Monocytes 2
Monocytes 3
Monocytes 4
Monocytes 5
NK cells
Oligodendrocytes
T cells
Ly6d
Cd79a
Igkc
Ighm
Jchain
H2-Aa
Arg1
Creb5
H2-Ab1
Ccl24
Enpp2
Mt3
1500015O10Rik
Clu
Ttr
Igfbp2
Bpgm
Hbb-bt
Hba-a2
Hba-a1
Hbb-bs
Retnlg
Ngp
Camp
S100a8
S100a9
Apoe
Cma1
Cpa3
Tpsb2
Mcpt2
Ccl2
H2-Eb1
Tgfbi
Cxcl9
Cd74
Cybb
Nr4a1
Napsa
Lyz2
Itgal
Gbp2
Ly6c2
Plac8
Ear2
Adgre4
Gngt2
Hist1h1b
Klrc2
Il2rb
Gzma
Ccl5
Ms4a4b
Trbc2
Cd3g
Cd3d
Cd3e

Figure S1. Single-cell and spatial transcriptomic analysis of the mouse GBM microenvironment, related to Figure 1. (A) Bioluminescence imaging of mice implanted with BTICs prior to tissue collection for scRNA-seq. (B) Schematic summarizing the number of mice used for single-cell RNA sequencing and spatial transcriptomics analyses. (C) UMAP plot displaying Seurat-defined clusters from scRNA-seq data. (D) InterCNV analysis of scRNA-seq of mouse GBM. (E) Pseudotime bar plot showing trajectory differences among monocyte and MDM subclusters. (F) UMAP visualization of pseudotime trajectories for monocytes and MDMs. (G) Pseudotime bar plot showing trajectory differences among microglia subclusters. (H) UMAP of pseudotime analysis for microglia, illustrating distinct differentiation trajectories. (I) Heatmap of DEGs across scRNA-seq clusters.

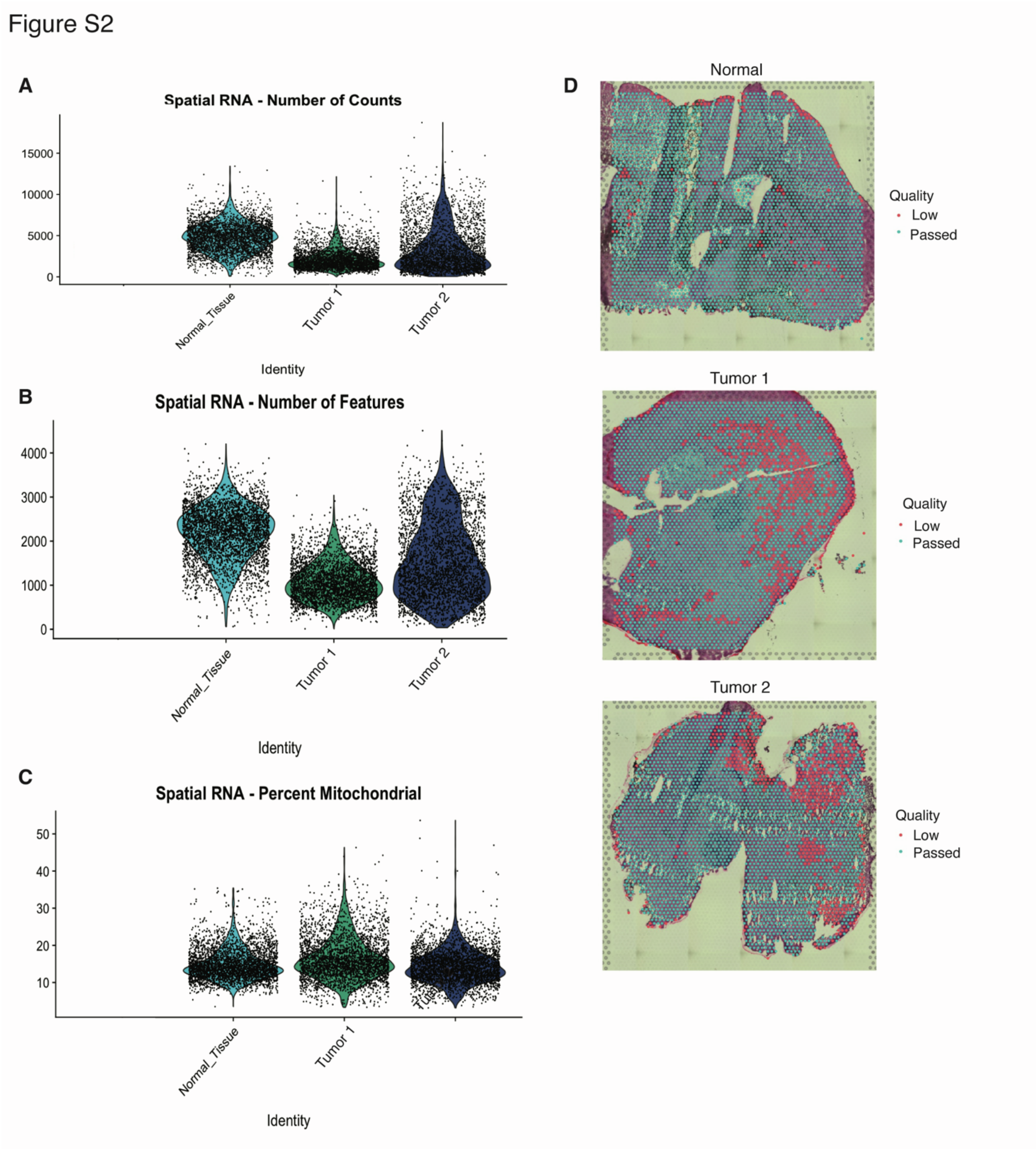

Figure S2. Quality control metrics of spatial transcriptomics in a mouse GBM model, related to Figure 1. Violin plots displaying (A) total UMI counts per spot (nCounts), (B) number of detected genes per spot (nFeatures), and (C) percentage of mitochondrial gene expression.

(D) Spatial feature plot showing spots that failed quality control (nCounts < 1000, nFeatures < 200, or mitochondrial percentage > 25%) versus those that passed the quality control thresholds.

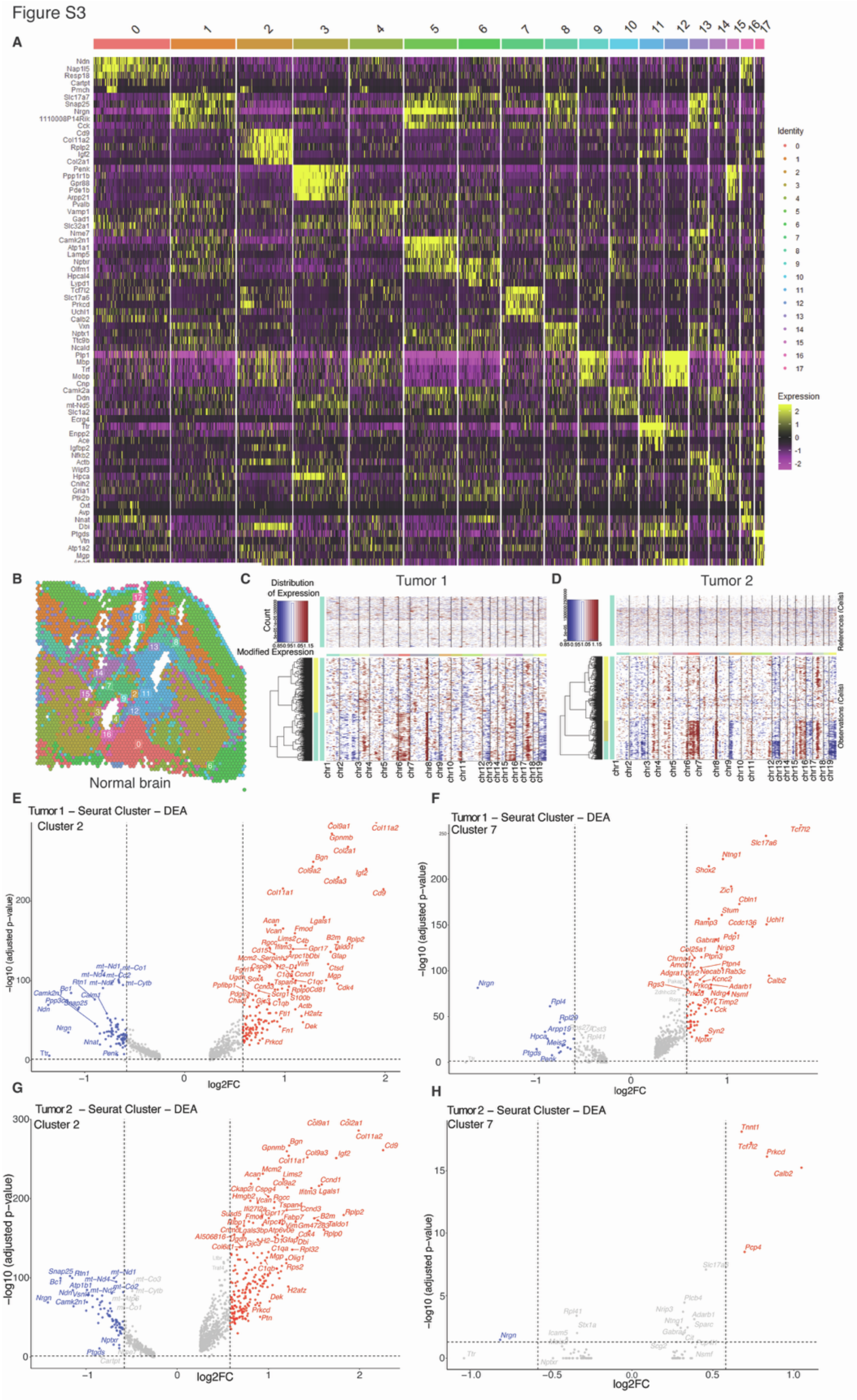

Figure S3. Spatial transcriptomic analysis of mouse GBM, related to Figure 1. (A) Heatmap of differentially expressed genes (DEGs) across spatial transcriptomics clusters. (B) Seurat-defined

spatial clusters overlaid on the tissue image of a normal mouse brain. (C and D) InferCNV plots for two tumor-bearing mice analyzed by spatial transcriptomics, indicating chromosomal alterations. (E–H) Volcano plots showing DEGs in cluster 2 (tumor region) and cluster 7 (tumor-adjacent region) from spatial transcriptomic profiling of mouse GBM.

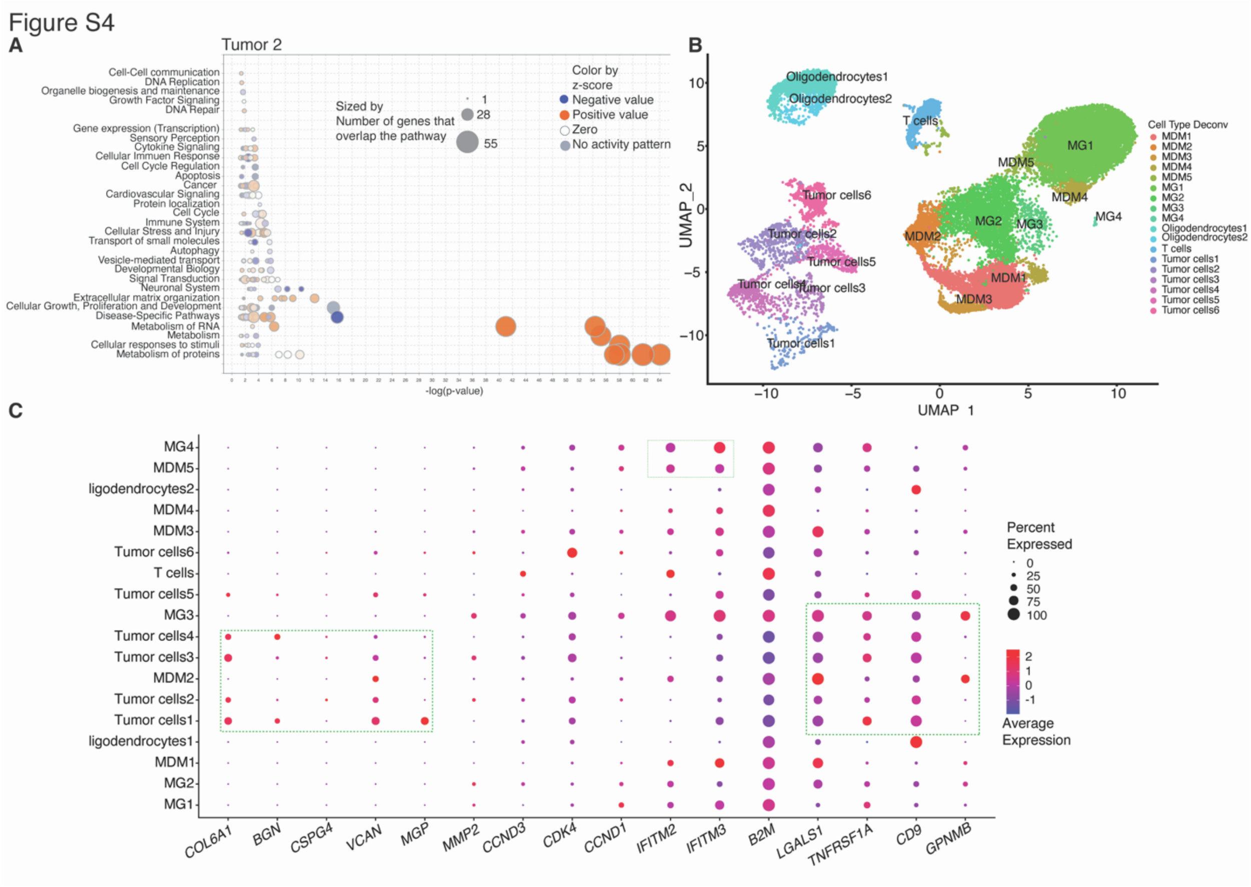

Figure S4. Transcriptomic profiling of the GBM microenvironment, related to Figure 1. (A) Canonical pathways identified by IPA enriched in DEGs between malignant and non-malignant regions based on spatial transcriptomics of mouse tumor 2. (B) UMAP plot showing Seurat clusters derived from scRNA-seq of human GBM. (C) Dot plot illustrating gene expression levels across cell types in the human TME using scRNA-seq data. Green dotted boxes highlight genes highly expressed in tumor cell clusters and immune cell clusters.

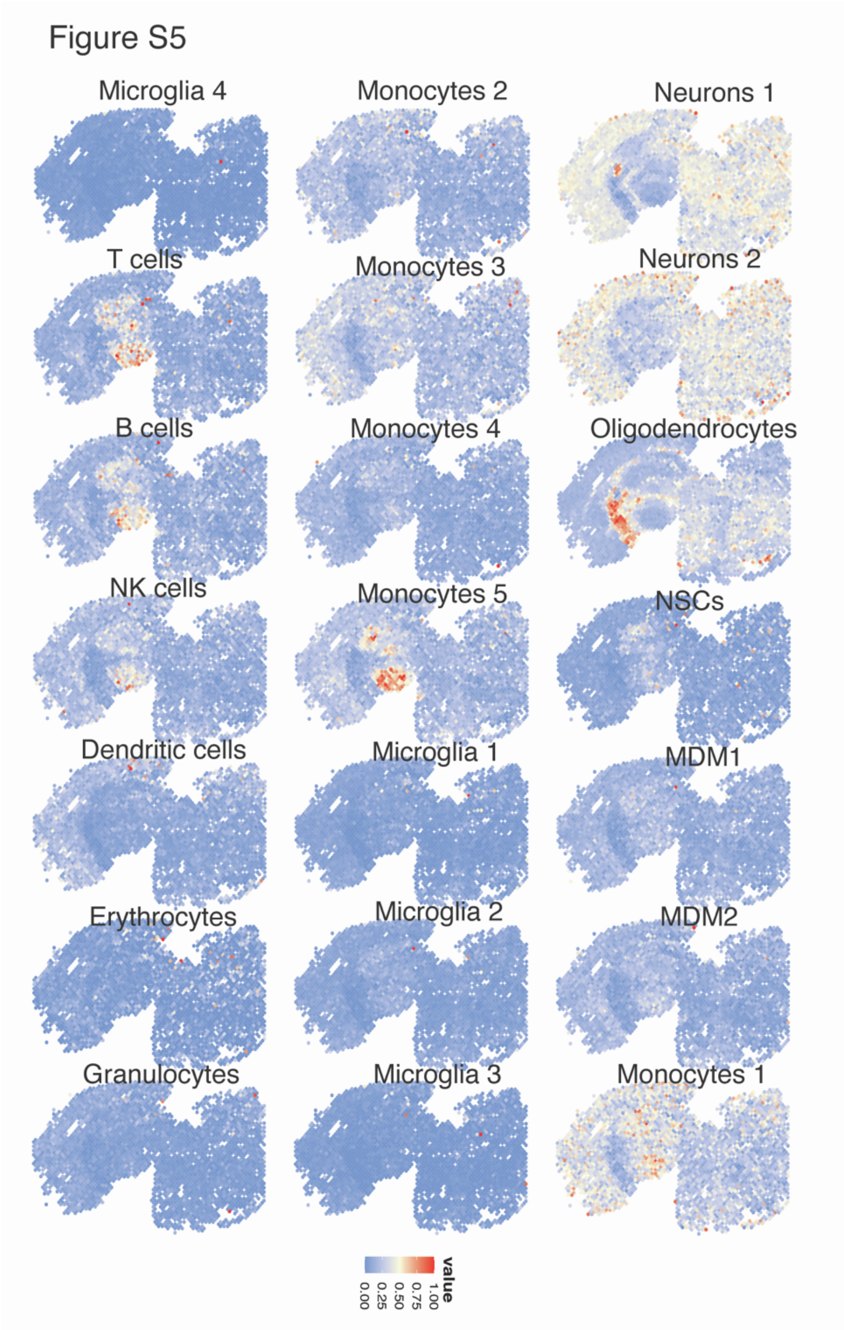

Figure S5. Spatial cellular composition in the brain of a mouse GBM, related to Figure 2. Deconvoluted cell type composition in the spatial transcriptomics data of mouse tumor 2, showing the distribution of distinct cell populations within the tumor microenvironment.

Figure S6

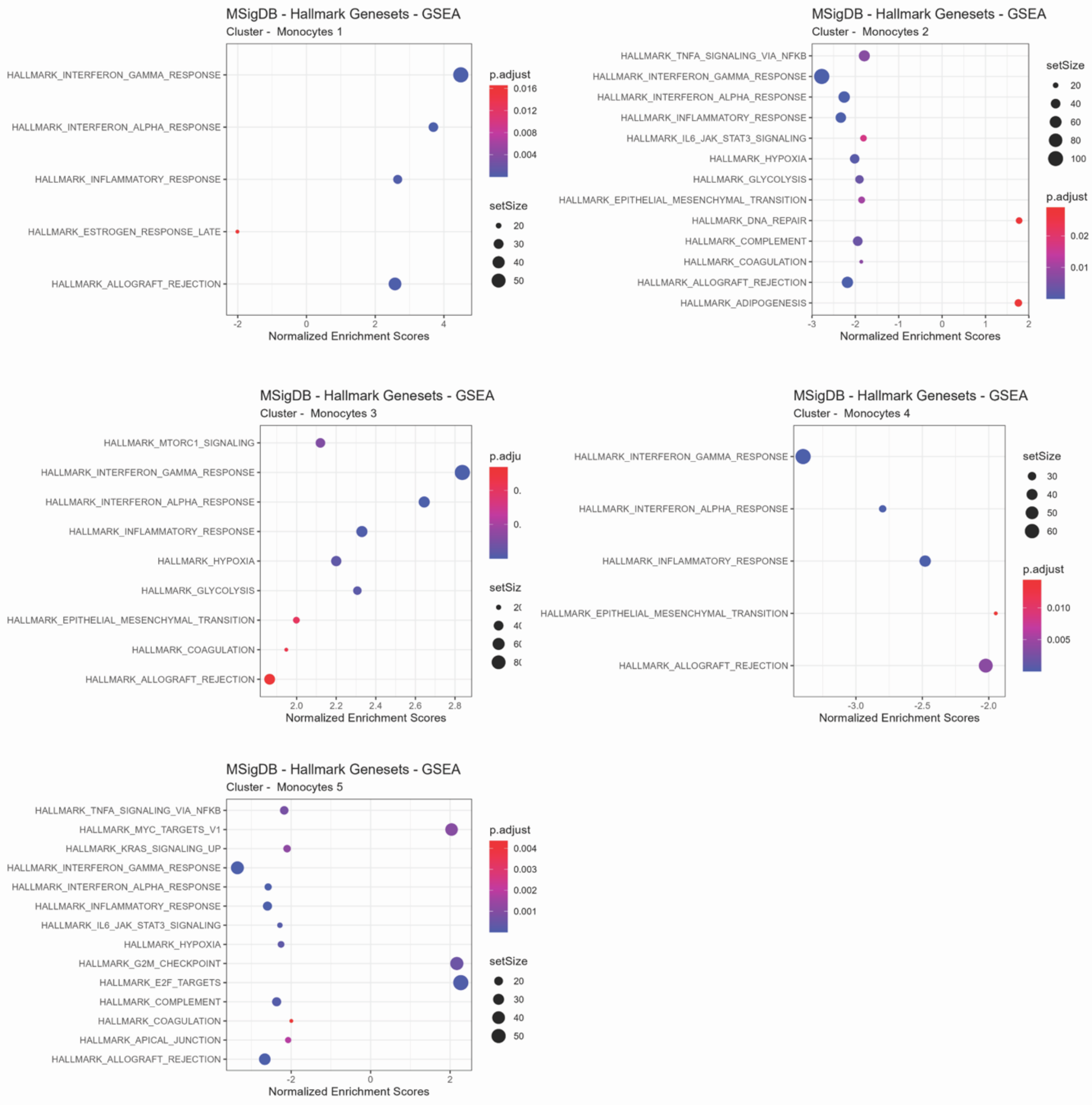

Figure S6. Gene set enrichment analysis of hallmark of cancer gene sets for monocyte subsets from scRNA-seq of mouse GBM, related to Figure 2.

Figure S7

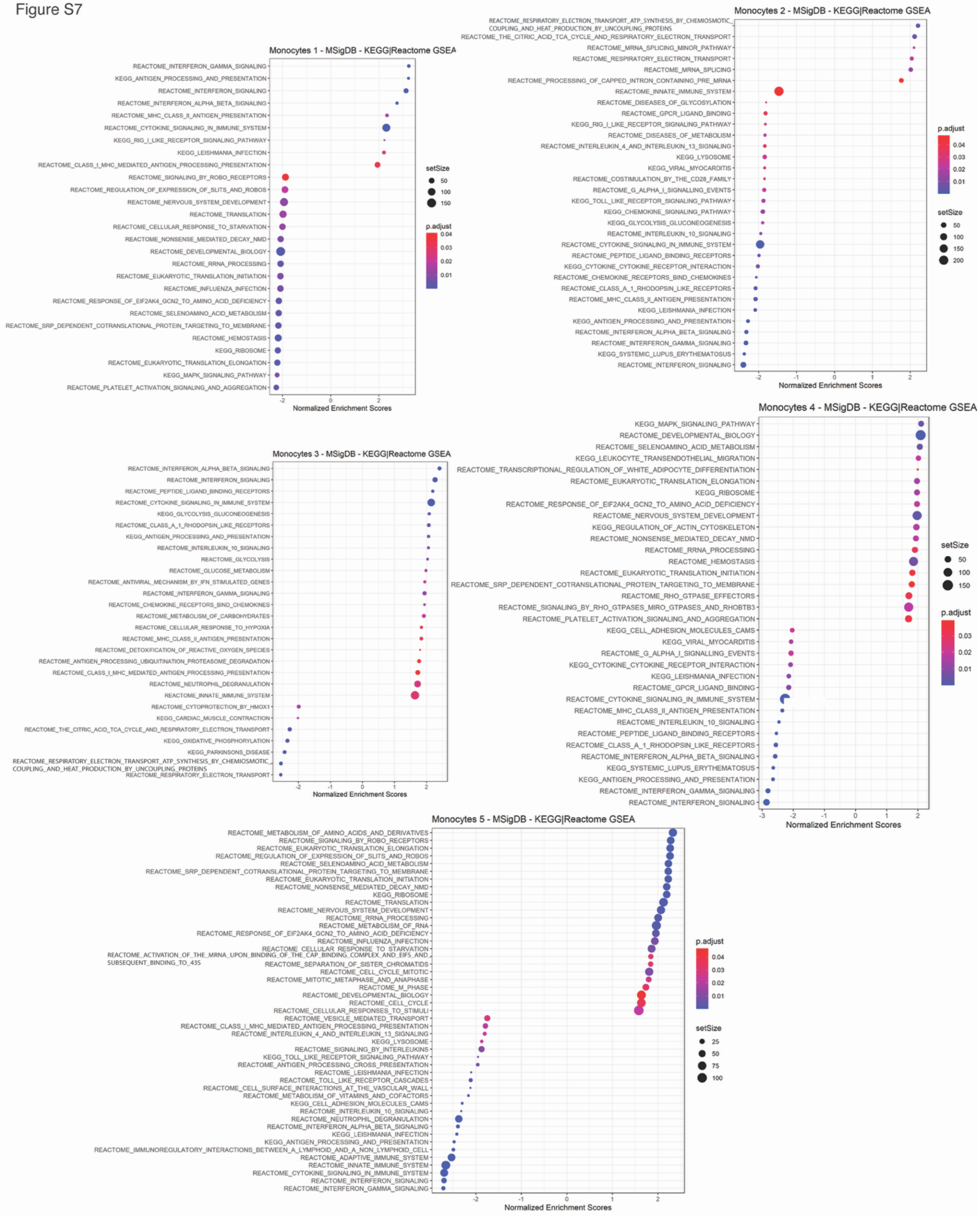

Monocytes 1 - MSigDB - KEGG|Reactome GSEA
REACTOME_INTERFERON_GAMMA_SIGNALING
KEGG_ANTIGEN_PROCESSING_AND_PRESENTATION
REACTOME_INTERFERON_SIGNALING
REACTOME_INTERFERON_ALPHA_BETA_SIGNALING
REACTOME_MHC_CLASS_II_ANTIGEN_PRESENTATION
REACTOME_CYTOKINE_SIGNALING_IN_IMMUNE_SYSTEM
KEGG_RIG_I_LIKE_RECEPTOR_SIGNALING_PATHWAY
KEGG_LEISHMANIA_INFECTION
REACTOME_CLASS_I_MHC_MEDIATED_ANTIGEN_PROCESSING_PRESENTATION
REACTOME_SIGNALING_BY_ROBO_RECEPTORS
REACTOME_REGULATION_OF_EXPRESSION_OF_SLITS_AND_ROBOS
REACTOME_NERVOUS_SYSTEM_DEVELOPMENT
REACTOME_TRANSLATION
REACTOME_CELLULAR_RESPONSE_TO_STARVATION
REACTOME_NONSENSE_MEDIATED_DECAY_NMD
REACTOME_DEVELOPMENTAL_BIOLOGY
REACTOME_RRNA_PROCESSING
REACTOME_EUKARYOTIC_TRANSLATION_INITIATION
REACTOME_INFLUENZA_INFECTION
REACTOME_RESPONSE_OF_EIF2AK4_GCN2_TO_AMINO_ACID_DEFICIENCY
REACTOME_SELENOAMINO_ACID_METABOLISM
REACTOME_SRP_DEPENDENT_COTRANSLATIONAL_PROTEIN_TARGETING_TO_MEMBRANE
REACTOME_HEMOSTASIS
KEGG_RIBOSOME
REACTOME_EUKARYOTIC_TRANSLATION_ELONGATION
KEGG_MAPK_SIGNALING_PATHWAY
REACTOME_PLATELET_ACTIVATION_SIGNALING_AND_AGGREGATION
-2 0 2
Normalized Enrichment Scores
setSize
50
100
150
p.adjust
0.04
0.03
0.02
0.01
Monocytes 2 - MSigDB - KEGG|Reactome GSEA
REACTOME_RESPIRATORY_ELECTRON_TRANSPORT_ATP_SYNTHESIS_BY_CHEMIOSMOTIC_COUPLING_AND_HEAT_PRODUCTION_BY_UNCOUPLING_PROTEINS
REACTOME_THE_CITRIC_ACID_TCA_CYCLE_AND_RESPIRATORY_ELECTRON_TRANSPORT
REACTOME_MRNA_SPLICING_MINOR_PATHWAY
REACTOME_RESPIRATORY_ELECTRON_TRANSPORT
REACTOME_MRNA_SPLICING
REACTOME_PROCESSING_OF_CAPPED_INTRON_CONTAINING_PRE_MRNA
REACTOME_INNATE_IMMUNE_SYSTEM
REACTOME_DISEASES_OF_GLYCOSYLATION
REACTOME_GPCR_LIGAND_BINDING
KEGG_RIG_I_LIKE_RECEPTOR_SIGNALING_PATHWAY
REACTOME_DISEASES_OF_METABOLISM
REACTOME_INTERLEUKIN_4_AND_INTERLEUKIN_13_SIGNALING
KEGG_LYSOSOME
KEGG_VIRAL_MYOCARDITIS
REACTOME_COSTIMULATION_BY_THE_CD28_FAMILY
REACTOME_G_ALPHA_I_SIGNALLING_EVENTS
KEGG_TOLL_LIKE_RECEPTOR_SIGNALING_PATHWAY
KEGG_CHEMOKINE_SIGNALING_PATHWAY
KEGG_GLYCOLYSIS_GLUCONEOGENESIS
REACTOME_INTERLEUKIN_10_SIGNALING
REACTOME_CYTOKINE_SIGNALING_IN_IMMUNE_SYSTEM
REACTOME_PEPTIDE_LIGAND_BINDING_RECEPTORS
KEGG_CYTOKINE_CYTOKINE_RECEPTOR_INTERACTION
REACTOME_CHEMOKINE_RECEPTORS_BIND_CHEMOKINES
REACTOME_CLASS_A_1_RHODOPSIN_LIKE_RECEPTORS
REACTOME_MHC_CLASS_II_ANTIGEN_PRESENTATION
KEGG_LEISHMANIA_INFECTION
KEGG_ANTIGEN_PROCESSING_AND_PRESENTATION
REACTOME_INTERFERON_ALPHA_BETA_SIGNALING
REACTOME_INTERFERON_GAMMA_SIGNALING
KEGG_SYSTEMIC_LUPUS_ERYTHEMATOSUS
REACTOME_INTERFERON_SIGNALING
-2 -1 0 1 2
Normalized Enrichment Scores
p.adjust
0.04
0.03
0.02
0.01
setSize
50
100
150
200
Monocytes 3 - MSigDB - KEGG|Reactome GSEA
REACTOME_INTERFERON_ALPHA_BETA_SIGNALING
REACTOME_INTERFERON_SIGNALING
REACTOME_PEPTIDE_LIGAND_BINDING_RECEPTORS
REACTOME_CYTOKINE_SIGNALING_IN_IMMUNE_SYSTEM
KEGG_GLYCOLYSIS_GLUCONEOGENESIS
REACTOME_CLASS_A_1_RHODOPSIN_LIKE_RECEPTORS
KEGG_ANTIGEN_PROCESSING_AND_PRESENTATION
REACTOME_INTERLEUKIN_10_SIGNALING
REACTOME_GLYCOLYSIS
REACTOME_GLUCOSE_METABOLISM
REACTOME_ANTIVIRAL_MECHANISM_BY_IFN_STIMULATED_GENES
REACTOME_INTERFERON_GAMMA_SIGNALING
REACTOME_CHEMOKINE_RECEPTORS_BIND_CHEMOKINES
REACTOME_METABOLISM_OF_CARBOHYDRATES
REACTOME_CELLULAR_RESPONSE_TO_HYPOXIA
REACTOME_MHC_CLASS_II_ANTIGEN_PRESENTATION
REACTOME_DETOXIFICATION_OF_REACTIVE_OXYGEN_SPECIES
REACTOME_ANTIGEN_PROCESSING_UBIQUITINATION_PROTEASOME_DEGRADATION
REACTOME_CLASS_I_MHC_MEDIATED_ANTIGEN_PROCESSING_PRESENTATION
REACTOME_NEUTROPHIL_DEGRANULATION
REACTOME_INNATE_IMMUNE_SYSTEM
REACTOME_CYTOPROTECTION_BY_HMOX1
KEGG_CARDIAC_MUSCLE_CONTRACTION
REACTOME_THE_CITRIC_ACID_TCA_CYCLE_AND_RESPIRATORY_ELECTRON_TRANSPORT
KEGG_OXIDATIVE_PHOSPHORYLATION
KEGG_PARKINSONS_DISEASE
REACTOME_RESPIRATORY_ELECTRON_TRANSPORT_ATP_SYNTHESIS_BY_CHEMIOSMOTIC_COUPLING_AND_HEAT_PRODUCTION_BY_UNCOUPLING_PROTEINS
REACTOME_RESPIRATORY_ELECTRON_TRANSPORT
-2 -1 0 1 2
Normalized Enrichment Scores
Monocytes 4 - MSigDB - KEGG|Reactome GSEA
KEGG_MAPK_SIGNALING_PATHWAY
REACTOME_DEVELOPMENTAL_BIOLOGY
REACTOME_SELENOAMINO_ACID_METABOLISM
KEGG_LEUKOCYTE_TRANSENDOTHELIAL_MIGRATION
REACTOME_TRANSCRIPTIONAL_REGULATION_OF_WHITE_ADIPOCYTE_DIFFERENTIATION
REACTOME_EUKARYOTIC_TRANSLATION_ELONGATION
KEGG_RIBOSOME
REACTOME_RESPONSE_OF_EIF2AK4_GCN2_TO_AMINO_ACID_DEFICIENCY
REACTOME_NERVOUS_SYSTEM_DEVELOPMENT
KEGG_REGULATION_OF_ACTIN_CYTOSKELETON
REACTOME_NONSENSE_MEDIATED_DECAY_NMD
REACTOME_RRNA_PROCESSING
REACTOME_HEMOSTASIS
REACTOME_EUKARYOTIC_TRANSLATION_INITIATION
REACTOME_SRP_DEPENDENT_COTRANSLATIONAL_PROTEIN_TARGETING_TO_MEMBRANE
REACTOME_RHO_GTPASE_EFFECTORS
REACTOME_SIGNALING_BY_RHO_GTPASES_MIRO_GTPASES_AND_RHOBTB3
REACTOME_PLATELET_ACTIVATION_SIGNALING_AND_AGGREGATION
KEGG_CELL_ADHESION_MOLECULES_CAMS
KEGG_VIRAL_MYOCARDITIS
REACTOME_G_ALPHA_I_SIGNALLING_EVENTS
KEGG_CYTOKINE_CYTOKINE_RECEPTOR_INTERACTION
KEGG_LEISHMANIA_INFECTION
REACTOME_GPCR_LIGAND_BINDING
REACTOME_CYTOKINE_SIGNALING_IN_IMMUNE_SYSTEM
REACTOME_MHC_CLASS_II_ANTIGEN_PRESENTATION
REACTOME_INTERLEUKIN_10_SIGNALING
REACTOME_PEPTIDE_LIGAND_BINDING_RECEPTORS
REACTOME_CLASS_A_1_RHODOPSIN_LIKE_RECEPTORS
REACTOME_INTERFERON_ALPHA_BETA_SIGNALING
KEGG_SYSTEMIC_LUPUS_ERYTHEMATOSUS
KEGG_ANTIGEN_PROCESSING_AND_PRESENTATION
REACTOME_INTERFERON_GAMMA_SIGNALING
REACTOME_INTERFERON_SIGNALING
-3 -2 -1 0 1 2
Normalized Enrichment Scores
setSize
50
100
150
p.adjust
0.03
0.02
0.01
Monocytes 5 - MSigDB - KEGG|Reactome GSEA
REACTOME_METABOLISM_OF_AMINO_ACIDS_AND_DERIVATIVES
REACTOME_SIGNALING_BY_ROBO_RECEPTORS
REACTOME_EUKARYOTIC_TRANSLATION_ELONGATION
REACTOME_REGULATION_OF_EXPRESSION_OF_SLITS_AND_ROBOS
REACTOME_SELENOAMINO_ACID_METABOLISM
REACTOME_SRP_DEPENDENT_COTRANSLATIONAL_PROTEIN_TARGETING_TO_MEMBRANE
REACTOME_EUKARYOTIC_TRANSLATION_INITIATION
REACTOME_NONSENSE_MEDIATED_DECAY_NMD
KEGG_RIBOSOME
REACTOME_TRANSLATION
REACTOME_NERVOUS_SYSTEM_DEVELOPMENT
REACTOME_RRNA_PROCESSING
REACTOME_METABOLISM_OF_RNA
REACTOME_RESPONSE_OF_EIF2AK4_GCN2_TO_AMINO_ACID_DEFICIENCY
REACTOME_INFLUENZA_INFECTION
REACTOME_CELLULAR_RESPONSE_TO_STARVATION
REACTOME_ACTIVATION_OF_THE_MRNA_UPON_BINDING_OF_THE_CAP_BINDING_COMPLEX_AND_EIFS_AND_SUBSEQUENT_BINDING_TO_43S
REACTOME_SEPARATION_OF_SISTER_CHROMATIDS
REACTOME_CELL_CYCLE_MITOTIC
REACTOME_MITOTIC_METAPHASE_AND_ANAPHASE
REACTOME_M_PHASE
REACTOME_DEVELOPMENTAL_BIOLOGY
REACTOME_CELL_CYCLE
REACTOME_CELLULAR_RESPONSES_TO_STIMULI
REACTOME_VESICLE_MEDIATED_TRANSPORT
REACTOME_CLASS_I_MHC_MEDIATED_ANTIGEN_PROCESSING_PRESENTATION
REACTOME_INTERLEUKIN_4_AND_INTERLEUKIN_13_SIGNALING
KEGG_LYSOSOME
REACTOME_SIGNALING_BY_INTERLEUKINS
KEGG_TOLL_LIKE_RECEPTOR_SIGNALING_PATHWAY
REACTOME_ANTIGEN_PROCESSING_CROSS_PRESENTATION
REACTOME_LEISHMANIA_INFECTION
REACTOME_TOLL_LIKE_RECEPTOR_CASCADES
REACTOME_CELL_SURFACE_INTERACTIONS_AT_THE_VASCULAR_WALL
REACTOME_METABOLISM_OF_VITAMINS_AND_COFACTORS
KEGG_CELL_ADHESION_MOLECULES_CAMS
REACTOME_INTERLEUKIN_10_SIGNALING
REACTOME_NEUTROPHIL_DEGRANULATION
REACTOME_INTERFERON_ALPHA_BETA_SIGNALING
KEGG_LEISHMANIA_INFECTION
KEGG_ANTIGEN_PROCESSING_AND_PRESENTATION
REACTOME_IMMUNOREGULATORY_INTERACTIONS_BETWEEN_A_LYMPHOID_AND_A_NON_LYMPHOID_CELL
REACTOME_ADAPTIVE_IMMUNE_SYSTEM
REACTOME_INNATE_IMMUNE_SYSTEM
REACTOME_CYTOKINE_SIGNALING_IN_IMMUNE_SYSTEM
REACTOME_INTERFERON_SIGNALING
REACTOME_INTERFERON_GAMMA_SIGNALING
-2 -1 0 1 2
Normalized Enrichment Scores
p.adjust
0.04
0.03
0.02
0.01
setSize
25
50
75
100

Figure S7. Gene set enrichment analysis of KEGG, and REACTOME for monocyte subsets from scRNA-seq of mouse GBM, related to Figure 2.

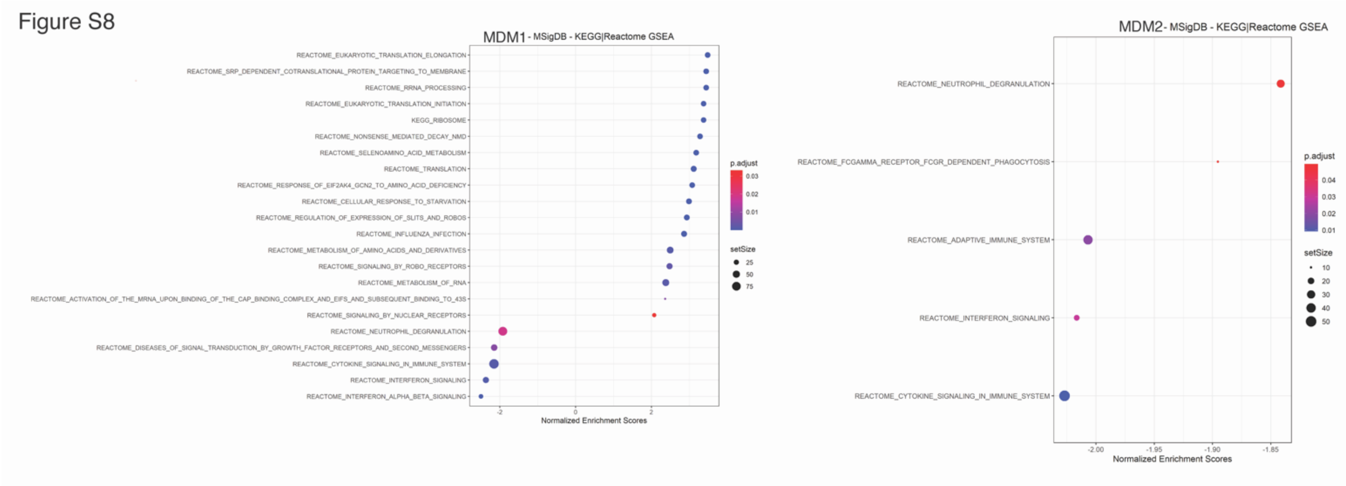

Figure S8. Gene set enrichment analysis of KEGG, and REACTOME for MDM subsets from scRNA-seq of mouse GBM, related to Figure 2.

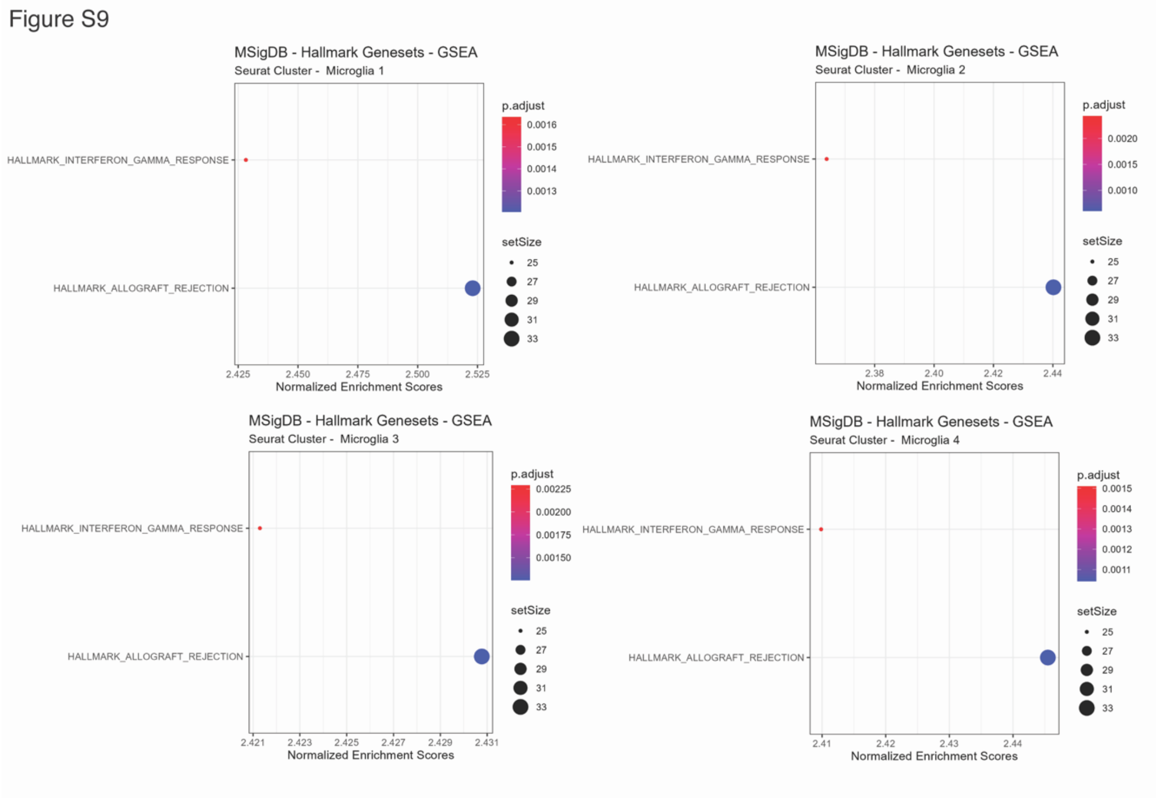

Figure S9. Gene set enrichment analysis of hallmark of cancer gene sets for microglia subsets from scRNA-seq of mouse GBM, related to Figure 2.

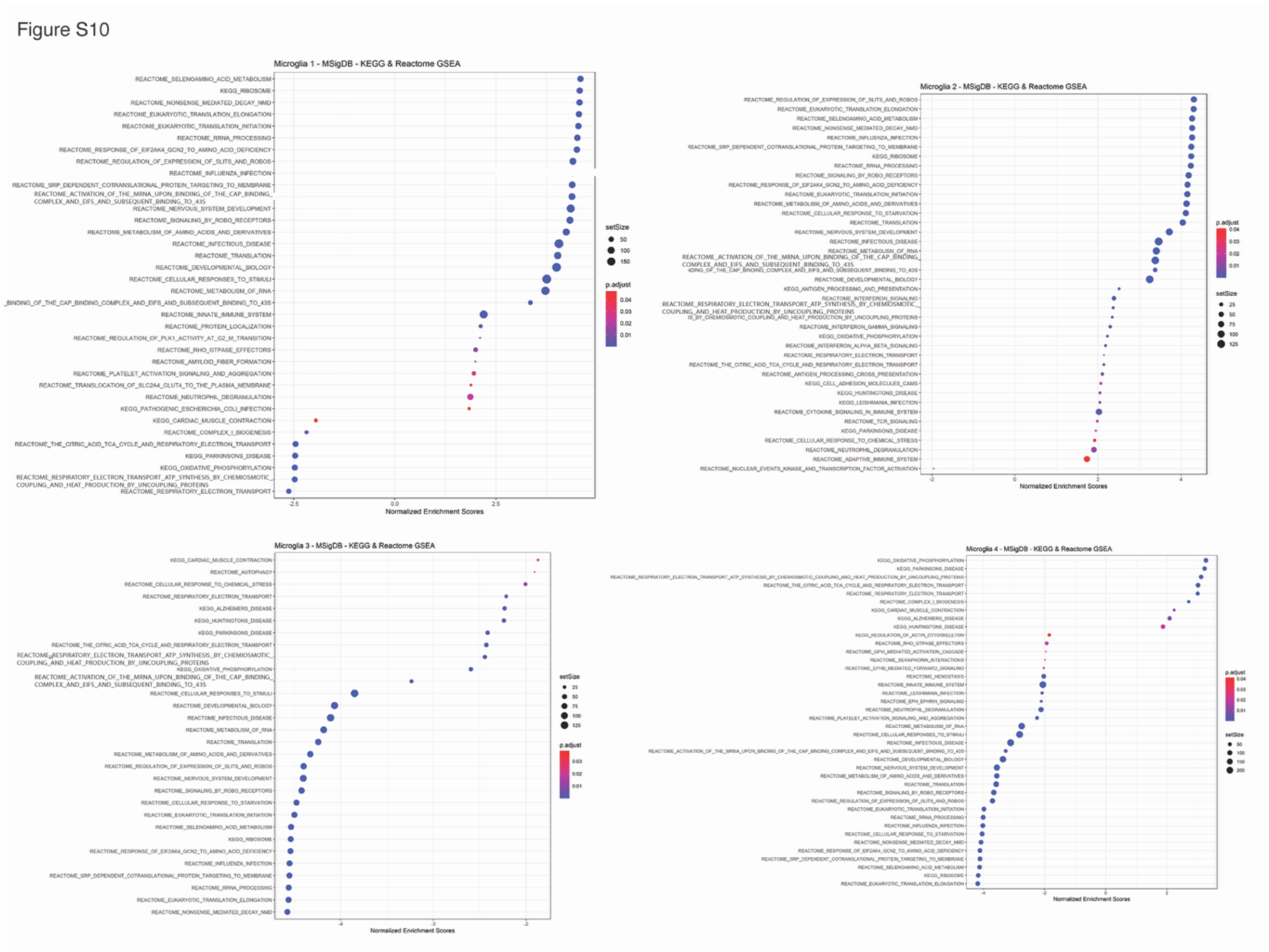

Figure S10. Gene set enrichment analysis of KEGG, and REACTOME for microglia subsets from scRNA-seq of mouse GBM, related to Figure 2.

Figure S11

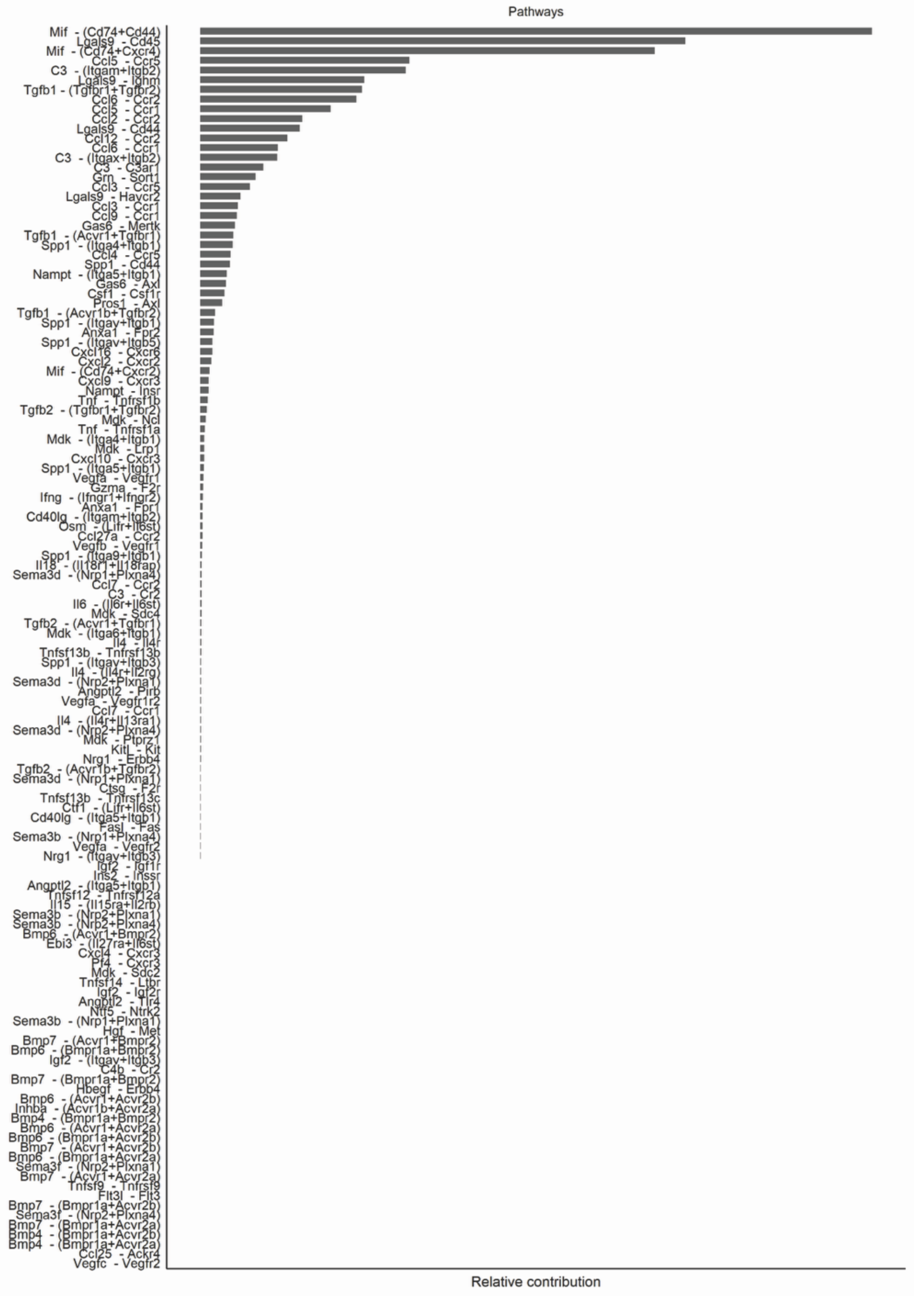

Figure S11. Signaling network in the GBM microenvironment, related to Figure 3. Contribution of each ligand-receptor pair to the overall signaling pathway calculated in scRNA-seq of mouse GBM.

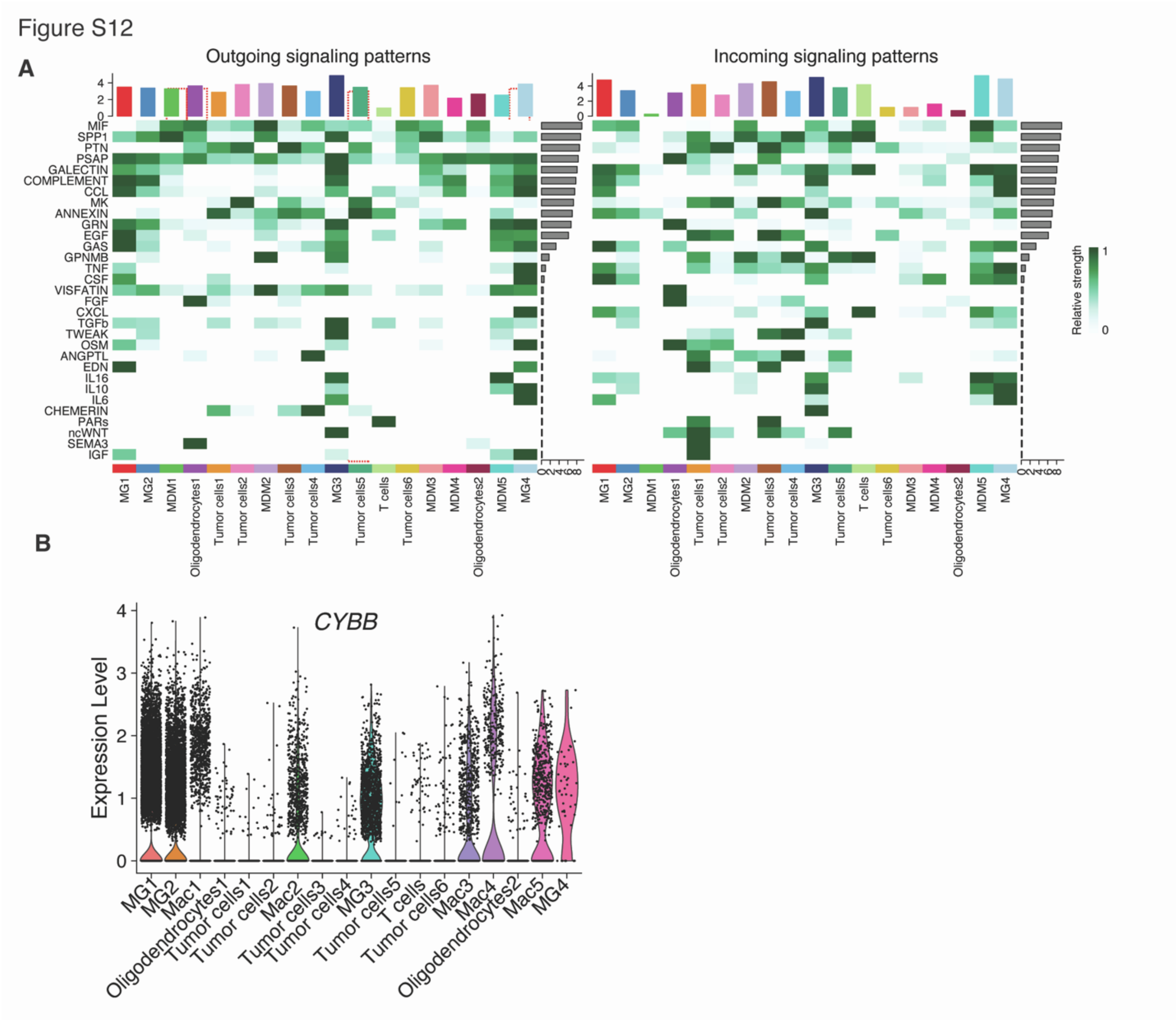

Figure S12. Signaling networks contributing to outgoing and incoming communication in human GBM, related to Figure 3 and 4. (A) Heatmaps displaying signaling pathways that contribute most to outgoing or incoming communication among specific cell groups in the human GBM scRNA-seq dataset. (B) Violin plot showing the expression levels of the *CYBB* gene across different cell clusters identified in the human GBM scRNA-seq data.

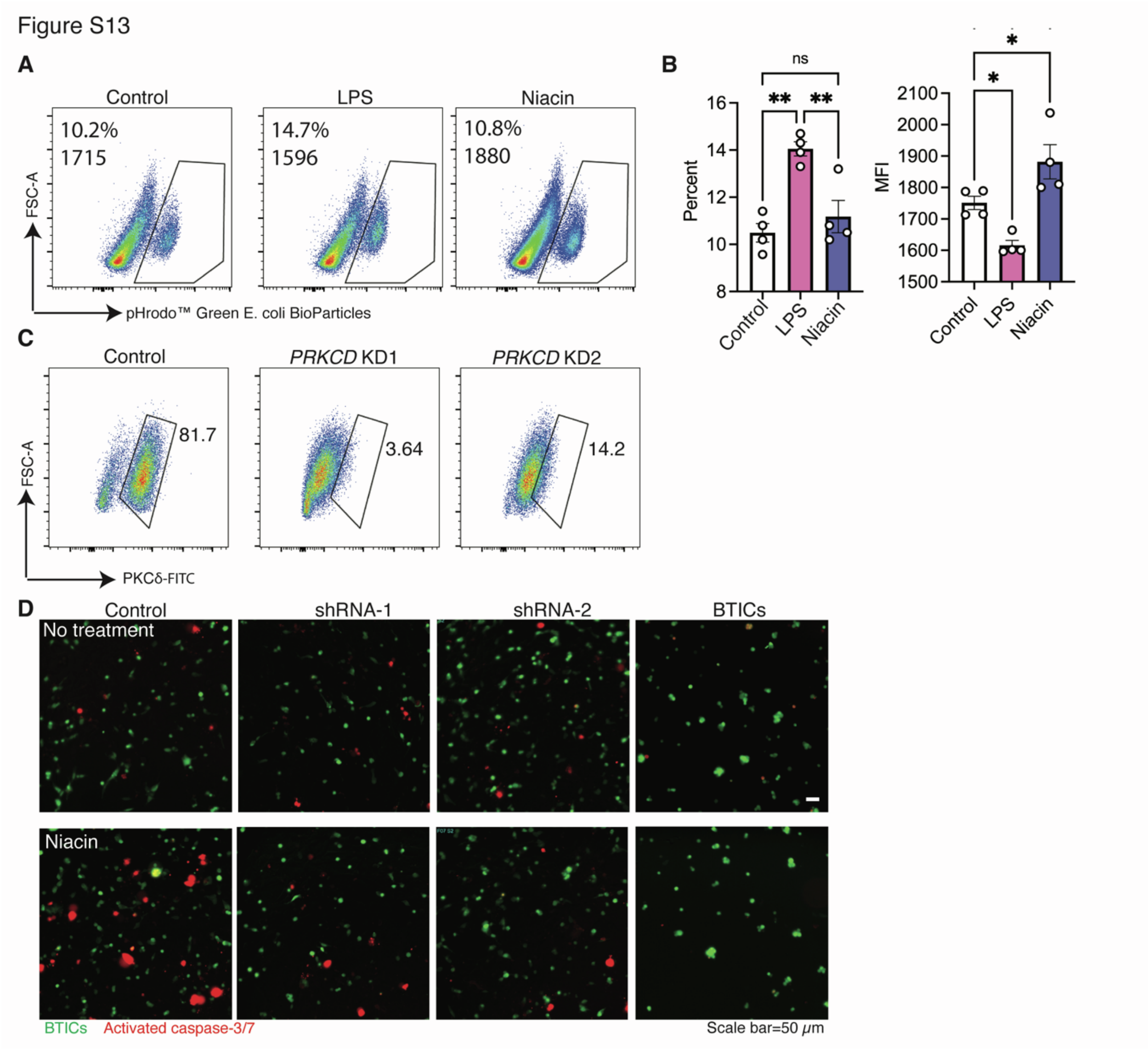

Figure S13. Involvement of PKCδ in the phagocytosis of tumor cells, related to Figure 5. (A and B) Representative flow cytometry plots and quantification of phagocytosis of pHrodo-labeled bioparticles by fetal human primary microglia treated with LPS and niacin. (C) Flow cytometry plots confirming downregulation of PKCδ using two different shRNAs in HMC3 cells. (D) Representative IF images from an apoptosis assay in a co-culture system of HMC3 cells with downregulated *PRKCD* expression and human BT025 cells. In panel B, statistical comparisons among multiple treatment groups were performed using one-way ANOVA followed by Benjamini–Hochberg correction.

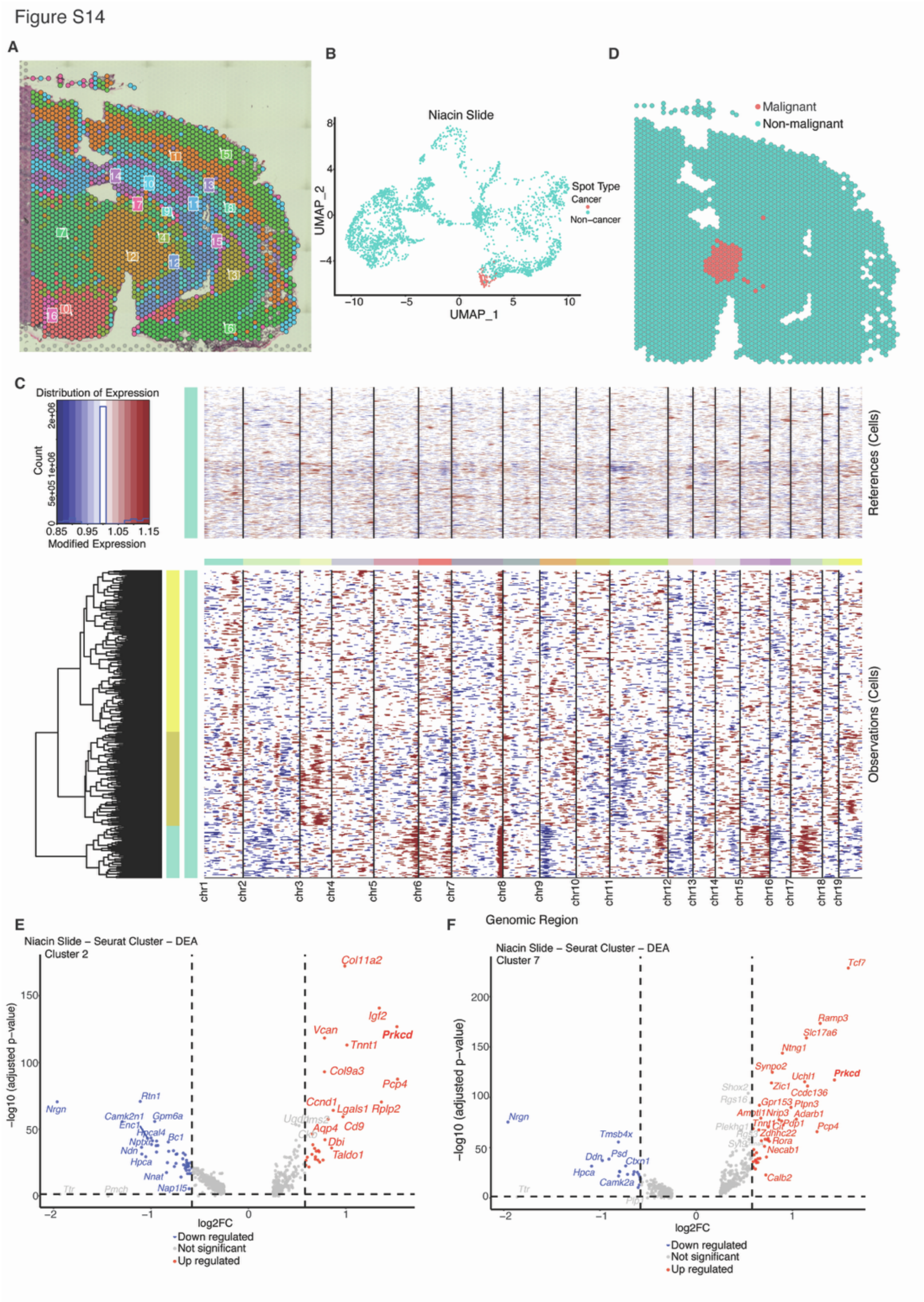

Figure S14. Spatial transcriptomic analysis of niacin-treated mouse brain, related to Figure 5. (A) Spatial DimPlot showing Seurat clusters overlaid on tissue images from tumor-bearing mice treated with niacin. (B) InferCNV analysis of spatial clusters identifying malignant and non-malignant spots within the tissue. (C) InferCNV heatmap displaying CNV events across chromosomes, with normal spots (top) and cancer spots (bottom). (D) Spatial map illustrating the distribution of malignant and non-malignant regions in the brain of a niacin-treated mouse. (E–F) Volcano plots showing DEGs between Seurat cluster 2 and cluster 7.

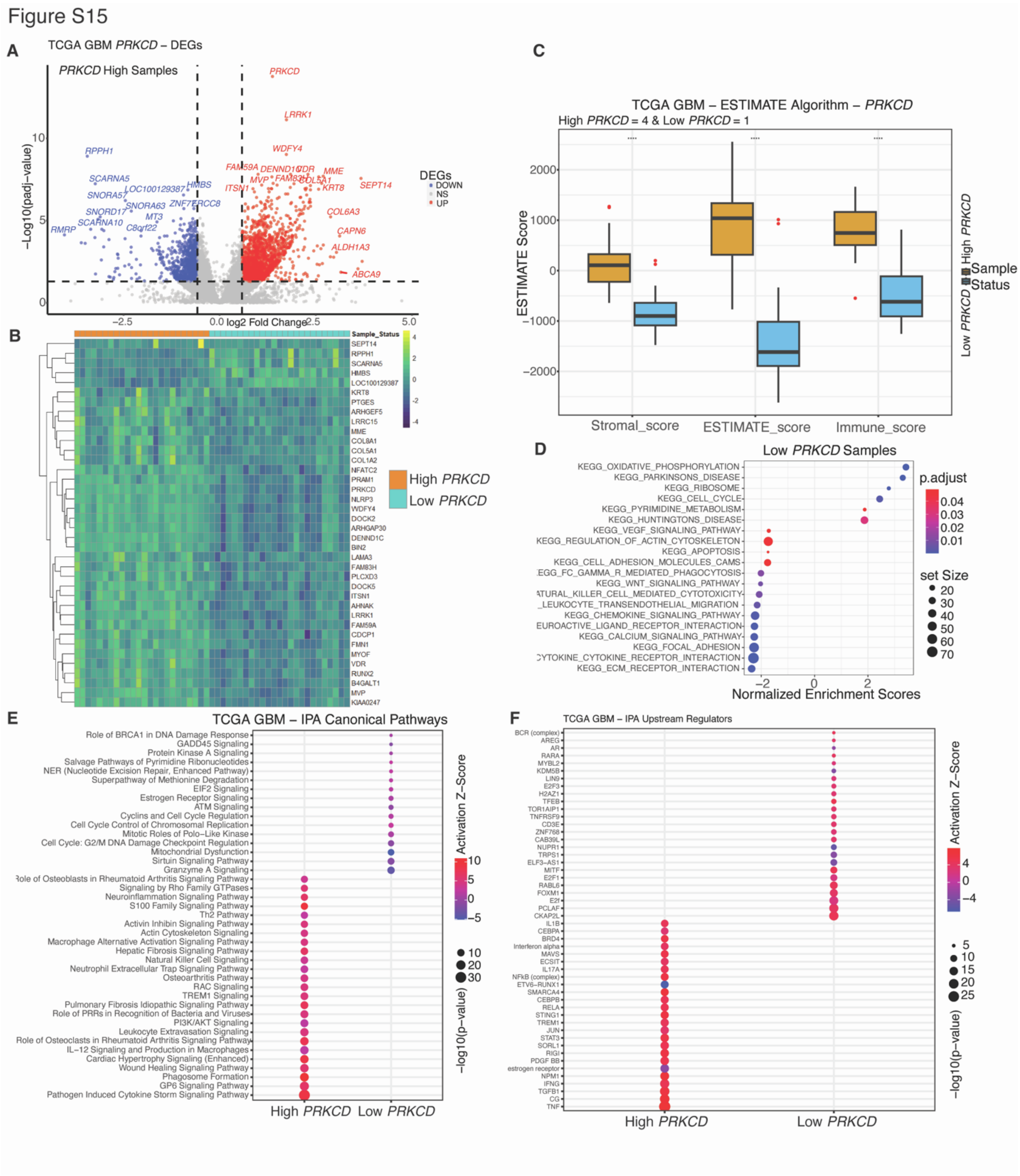

Figure S15. Analysis of *PRKCD* expression in TCGA GBM dataset, related to Figure 6. (A) Volcano plot showing differentially expressed genes (DEGs) between PRKCD-high and *PRKCD*-low samples in the TCGA GBM RNA-seq dataset. (B) Heatmap of top DEGs illustrating differences in gene expression between the two groups. (C) ESTIMATE analysis comparing immune, stromal, and tumor purity scores between *PRKCD*-high and PRKCD-low samples; significance determined by Wilcoxon rank-sum test ($p < 0.05$). (D) GSEA and MSigDB

analyses identifying enriched KEGG and REACTOME pathways in *PRKCD*-low samples. (E–F) Dot plots showing Ingenuity Pathway Analysis (IPA) results for canonical pathways and upstream regulators enriched in either *PRKCD*-high or *PRKCD*-low samples.
